# Integration of epigenomic and transcriptomic profiling uncovers EZH2 target genes linked to cysteine metabolism in hepatocellular carcinoma

**DOI:** 10.1038/s41419-024-07198-0

**Published:** 2024-11-08

**Authors:** Jaehyun Lee, Chaelin You, Geunho Kwon, Junho Noh, Kyubin Lee, Kyunghwan Kim, Keunsoo Kang, Kyuho Kang

**Affiliations:** 1 Department of Biological Sciences and Biotechnology, Chungbuk National University, Cheongju, Korea; 2 Department of Microbiology, College of Science & Technology, Dankook University, Cheonan, Korea

**Keywords:** Liver cancer, Liver cancer

## Abstract

Enhancer of zeste homolog 2 (EZH2), a key protein implicated in various cancers including hepatocellular carcinoma (HCC), is recognized for its association with epigenetic dysregulation and pathogenesis. Despite clinical explorations into EZH2-targeting therapies, the mechanisms underlying its role in gene suppression in HCC have remained largely unexplored. Here, we integrate epigenomic and transcriptomic analyses to uncover the transcriptional landscape modulated by selective EZH2 inhibition in HCC. By reanalyzing transcriptomic data of HCC patients, we demonstrate that *EZH2* overexpression correlates with poor patient survival. Treatment with the EZH2 inhibitor tazemetostat restored expression of genes involved in cysteine-methionine metabolism and lipid homeostasis, while suppressing angiogenesis and oxidative stress-related genes. Mechanistically, we demonstrate EZH2-mediated H3K27me3 enrichment at *cis*-regulatory elements of transsulfuration pathway genes, which is reversed upon inhibition, leading to increased chromatin accessibility. Among 16 EZH2-targeted candidate genes, *BHMT* and *CDO1* were notably correlated with poor HCC prognosis. Tazemetostat treatment of HCC cells increased *BHMT* and *CDO1* expression while reducing levels of ferroptosis markers *FSP1*, *NFS1*, and *SLC7A11*. Functionally, EZH2 inhibition dose-dependently reduced cell viability and increased lipid peroxidation in HCC cells. Our findings reveal a novel epigenetic mechanism controlling lipid peroxidation and ferroptosis susceptibility in HCC, providing a rationale for exploring EZH2-targeted therapies in this malignancy.

## Introduction

Hepatocellular carcinoma (HCC), the predominant form of primary liver cancer, accounts for nearly 830,000 cancer-associated mortalities globally every year [1, 2]. While a decline in HCC related to viral infections has been achieved through vaccination, the rise of non-alcoholic steatohepatitis (NASH) and metabolic syndrome signals an alarming increase in HCC cases [3–5]. The multifaceted development of HCC involves cellular alterations such as senescence, mutations, and epigenetic modifications within a necro-inflammatory context, leading to tumorigenesis. As insights emerge that aberrations or genetic variations in epigenetic modulators can broadly influence cellular biology, culminating in cancer development [6], epigenetic interventions are emerging as a promising therapeutic strategy for HCC [7, 8]. However, the intricate role of epigenetic modulators in the evolution of HCC requires more extensive exploration to define the optimal strategies for targeting these essential components in combating this challenging disease.

Enhancer of zeste homolog 2 (EZH2), the catalytic core of the polycomb repressive complex 2 (PRC2), is fundamental to gene regulation, particularly through its central role in tri-methylating histone 3 lysine 27 (H3K27me3) to suppress gene expression. Remarkably, overexpression of EZH2 has been identified in various cancers, including HCC, where it exhibits a positive correlation with tumor malignancy [9–11]. Within HCC, the roles of EZH2 extend to the inhibition of NK cell-mediated anti-tumor immunity and the promotion of both carcinogenesis and drug resistance [12–15]. These observations reinforce the perspective that targeting EZH2 could present a valuable therapeutic path for HCC management. The selective EZH2 inhibitor, tazemetostat, has garnered FDA approval for treating epithelioid sarcoma and follicular lymphoma [16–18]. Its efficacy is currently under investigation in various solid tumors and cases of advanced cancer with hepatic impairment, as evidenced by the clinical trials (NCT01897571, NCT04241835, and NCT05023655). As evaluations of tazemetostat continue, the comprehensive mapping of EZH2’s target genes in HCC remains a work in progress [16, 19, 20]. Investigating the genes affected by EZH2 inhibition and deciphering the complex mechanisms involved may illuminate the therapeutic possibilities of this targeted strategy in HCC.

Elevated cysteine levels have been implicated in an increased risk of various disorders, notably atherosclerosis and chronic liver disease [21–23]. Within the complex domain of cancer biology, cysteine metabolism has been recognized as a significant determinant in shaping therapeutic resistance and cellular responses to hypoxia and various stressors [24–27]. Such insights have driven the exploration of therapeutic approaches focusing on cysteine metabolism in cancer cells. Enzymes such as betaine-homocysteine thiol methyltransferase (BHMT) and cysteine dioxygenase 1 (CDO1) have been pinpointed as vital actors in this regard, with connections to hepatic health [28, 29]. A decline in BHMT expression has been observed in hepatocellular carcinoma (HCC), suggesting its potential as a marker for fatty liver disease and HCC onset [30, 31]. A recent study has shown that a lack of BHMT can lead to hepatocarcinogenesis and tumor growth by increasing the activity of glucose-6-phosphate dehydrogenase (G6PD) and pentose phosphate pathway (PPP) metabolism in HCC [32]. The *CDO1* gene, a tumor suppressor, is often silenced through promoter methylation across various human cancers. It plays a role in reducing cancer cell proliferation by inducing oxidative stress in gastric cancer [33, 34]. Additionally, the involvement of CDO1 in tumorigenesis in HCC is emphasized by gene silencing and promoter methylation, suggesting its utility as a biomarker for HBV-related HCC [35]. Recently, a study found that TRIM47 promotes the ubiquitination of CDO1, leading to a reduction in protein levels, and it can regulate ferroptosis in HCC [36]. The regulation of these sulfur amino acid (SAA) enzymes by HNF4α in liver cancer has drawn attention, with the intriguing observation that HNF4α, typically a tumor suppressor, may adopt a pro-tumor stance by inhibiting erastin-induced ferroptosis when its equilibrium is offset by hypermethylated in cancer 1 (HIC1) [37, 38]. The multifaceted interplay between cysteine metabolism and HNF4α warrants deeper exploration, offering avenues for precision therapeutic advancements.

Ferroptosis, distinct from apoptosis, autophagy, necroptosis, and pyroptosis, is a regulated cell death mechanism governed by cellular iron accumulation and subsequent reactive oxygen species (ROS) production, leading to lipid peroxidation within cellular membranes [39, 40]. The cysteine-glutathione-peroxidase 4 (GPX4) signaling pathway serves as a protective barrier against ferroptosis, whereas inhibiting the cystine uptake transporter, SLC7A11, promotes ferroptosis [40–42]. Notably, Sorafenib, a key therapeutic in HCC management, is known to trigger ferroptosis by targeting SLC7A11 [43–45]. However, the precise epigenetic mechanisms of *SLC7A11* gene regulation in HCC remains elusive. Adding to this intricacy, CDO1 activation has been spotlighted as a pro-ferroptotic factor in gastric cancers, depleting intracellular cysteine and intensifying lipid peroxidation and ROS generation [46, 47]. These findings highlight cysteine-methionine metabolism as a potential therapeutic target in HCC. Yet, the precise mechanisms orchestrating this metabolism within HCC are not fully delineated, underscoring the need for deeper investigation to unlock its therapeutic potential.

In this study, we aimed to identify distinct target genes in HCC whose deregulation can be ascribed to EZH2-mediated H3K27me3 modifications. Employing bulk RNA-seq and ATAC-seq on human HCC cell lines, we unveiled pronounced gene upregulation within the cysteine-methionine metabolic pathway following EZH2 inhibition. Notably, *BHMT* and *CDO1* demonstrated dramatic changes in chromatin accessibility and were markedly derepressed. Single-cell RNA sequencing (scRNA-seq) further illuminated that *BHMT* and *CDO1* were uniquely expressed in normal hepatocytes, but not in other cell types. Analysis of transcriptomic datasets from various HCC cohorts indicated diminished expression of *BHMT* and *CDO1*, associated with adverse clinical outcomes. Additionally, our findings revealed that EZH2 inhibition modulates the negative ferroptosis markers *SLC7A11, NFS1*, and *FSP1*. Collectively, our study illuminates distinct transcriptional changes triggered by EZH2 inhibition, suggesting a pivotal role in promoting lipid ROS accumulation, potentially leading to ferroptotic cell death in HCC.

## Results

### Elevated EZH2 expression correlates with unfavorable prognosis in HCC patients

**Fig. 1:**
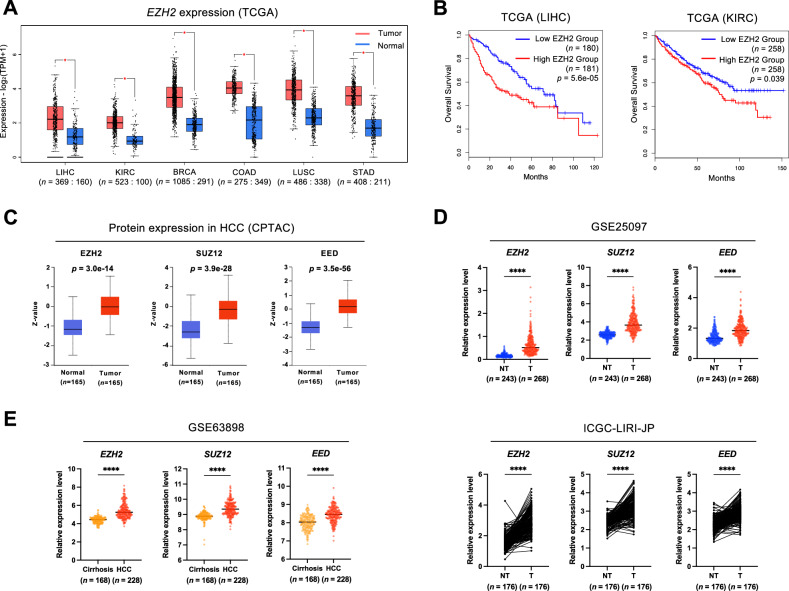
Higher expression of *EZH2* is associated with poor prognosis in HCC. **A** *EZH2* expression levels in LIHC, KIRC, BRCA, COAD, LUSC, and STAD compared to corresponding controls in the TCGA cohort. **B** Kaplan–Meier survival plots showing overall survival with high vs low expression of *EZH2* in liver and kidney cancer. Patients were divided into high (50%) or low (50%) expression groups. **C**–**E** Protein or RNA expression levels of *EZH2*, *SUZ12,* and *EED* were overexpressed in HCC tumors compared to non-tumor or cirrhosis tissues in four independent cohorts. *P* values were determined by unpaired *t*-test (*p < 0.05, **p < 0.01, ***p < 0.001, ****p < 0.0001).

To elucidate the correlation between *EZH2* expression and survival outcomes in different tumor patients, we examined six prominent cancer types (liver hepatocellular carcinoma [LIHC], kidney renal clear cell carcinoma [KIRC], breast invasive carcinoma [BRCA], colon adenocarcinoma [COAD], lung squamous cell carcinoma [LUSC], stomach adenocarcinoma [STAD]) within the cancer genome atlas (TCGA). A pronounced increase in *EZH2* expression was observed in tumor tissues relative to normal counterparts for all six cancer types (Fig. 1A). Employing Kaplan-Meier survival analysis, we classified the survival outcomes into two groups: unfavorable and non-significant, based on *EZH2* overexpression. Notably, LIHC and KIRC patient groups with elevated *EZH2* expression exhibited significantly reduced OS compared to those with lower *EZH2* expression. In contrast, the BRCA, COAD, LUSC, and STAD patient groups did not display a discernible correlation between *EZH2* expression levels and survival rates (Fig. 1B and Supplementary Fig. 1A). Subsequently, we leveraged proteomic data from the CPTAC database to validate EZH2 expression at the protein level, finding marked overexpression of the EZH2 protein in LIHC tumor tissues, though not in KIRC (Fig. 1C and Supplementary Fig. 1B).

Our focus then shifted to the specific role of EZH2 in HCC. Using transcriptome data from independent HCC cohorts (GSE25097, ICGC-LIRI-JP, and GSE14520), we ascertained that EZH2 was distinctly elevated across all HCC cohorts, accompanied by significant upregulation of the PRC2 complex subunits *SUZ12* and *EED*, of which EZH2 is a constituent (Fig. 1D and Supplementary Fig. 1C). Overexpression of SUZ12 and EED protein was also detected in KIRC tumor tissues, albeit to a lesser extent than in LIHC (Fig. 1C and Supplementary Fig. 1B). Intriguingly, a subsequent analysis using the GSE63898 cohort reaffirmed that PRC2 subunits inclusive of EZH2 displayed a higher expression in HCC than in cirrhosis, often considered an HCC precursor state (Fig. 1E). Overall, these results demonstrate that higher expression of EZH2 is intimately associated with adverse clinical outcomes in HCC.

### EZH2 inhibition reverses the gene expression program by reducing H3K27me3 levels

**Fig. 2:**
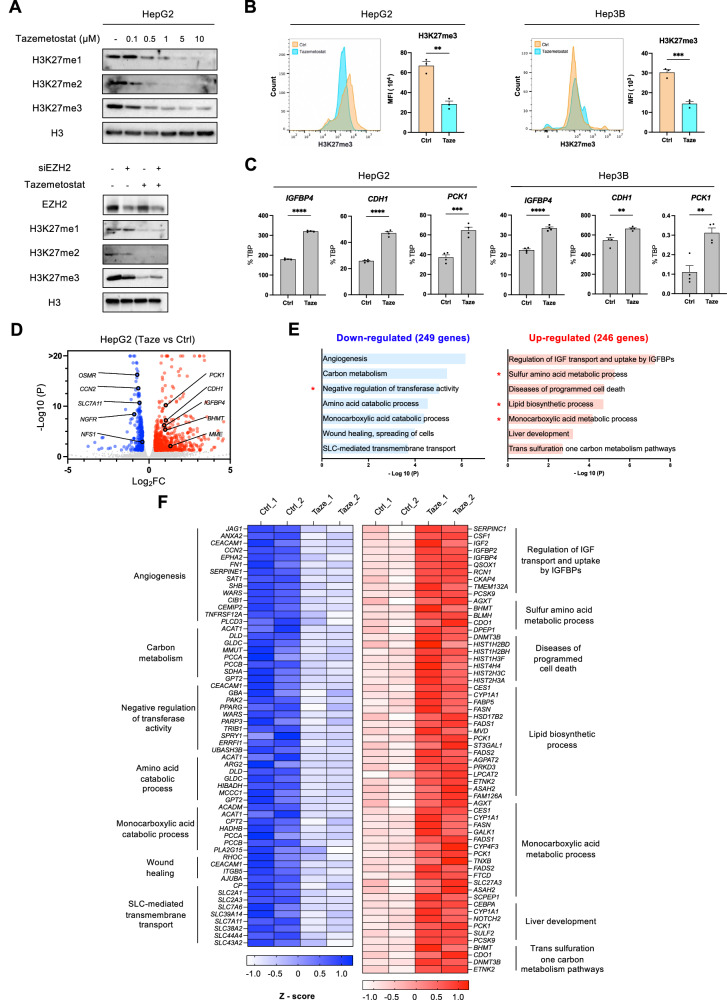
EZH2 inhibition reverses the gene expression program by decreasing H3K27me3. **A** Western blot analysis of EZH2 and H3K27 methylation levels in wild-type and EZH2-knockdown HepG2 cells treated with tazemetostat. **B** Histogram showing the levels of H3K27me3 in HepG2 and Hep3B cells treated with tazemetostat (Taze, light blue) or DMSO (Ctrl, orange). The number of cells and median fluorescent intensity (MFI) are shown (*n* = 3). **C** Transcript levels of *IGFBP4*, *CDH1*, and *PCK1* treated with tazemetostat for 48 h in HepG2 and 96 h in Hep3B (*n* = 3). **D** Volcano plot of transcriptomic changes between control and tazemetostat-treated cells, with colored dots representing genes with significant (FDR < 0.05) and greater than 1.3-fold expression changes. **E**, **F** Biological pathways of DEGs using Metascape and heatmap of GO terms related to up- or downregulated gene sets in response to EZH2 inhibition. *TBP* was used for the normalization of each cell line level. *P* values were determined by unpaired *t*-test (*p < 0.05, **p < 0.01, ***p < 0.001, ****p < 0.0001).

To assess the effect of EZH2 inhibition on H3K27me3 levels, we treated HepG2 HCC cells with tazemetostat, a selective EZH2 inhibitor, or performed EZH2 knockdown. Western blot analysis revealed a marked reduction in H3K27 mono-, di-, and tri-methylation levels following both tazemetostat treatment and siRNA-mediated EZH2 knockdown (Fig. 2A). The global reduction of H3K27me3 levels upon tazemetostat treatment in both HepG2 and Hep3B HCC cell lines was confirmed by flow cytometry (Fig. 2B). To further confirm if EZH2 target genes are indeed derepressed through H3K27me3 reduction, we evaluated gene expression levels by RT-qPCR. EZH2 inhibition restored *IGFBP4*, a tumor suppressor gene typically downregulated by EZH2 in HCC [14]. Similarly, *CDH1* and *PCK1*, known to be suppressed in HCC [48–50], showed marked upregulation in both tazemetostat-treated HCC cell lines (Fig. 2C).

To gain a comprehensive understanding of the transcriptional dynamics induced by the inhibition of EZH2-mediated H3K27me3, we performed RNA sequencing on an HCC cell line treated with tazemetostat, as well as with EZH2 knockdown (Supplementary Fig. 2A). Our examination focused on 495 differentially expressed genes (DEGs), comprising 246 upregulated and 249 downregulated genes (FDR < 0.05, 1.3-fold difference in expression, and TPM > 1.5) when comparing the transcriptomic profiles of tazemetostat-treated and untreated cells (Fig. 2D). A subset of these DEGs, influenced by the cessation of EZH2 activity, is highlighted in the volcano plot (Fig. 2D). Consistent with the RT-qPCR observations, our RNA-seq findings validated the significant derepression of *IGFBP4, CDH1*, and *PCK1* upon EZH2 inhibition (Supplementary Fig. 2B). Additionally, RNA-seq analysis from EZH2 knockdown experiments, directly targeting *EZH2* expression, identified 843 upregulated and 646 downregulated genes (Supplementary Fig. 2C, D). We found that 54 upregulated and 39 downregulated genes were regulated by both tazemetostat and EZH2 knockdown (Supplementary Fig. 2F).

To elucidate the influence of EZH2-mediated H3K27me3 on biological pathways, we conducted a GO analysis using DEGs. Our analysis revealed that genes associated with metabolic pathways, including sulfur amino acid and monocarboxylic acid metabolism, as well as lipid biosynthetic processes, were predominantly increased by tazemetostat treatment and EZH2 knockdown (Fig. 2E and Supplementary Fig. 2E). In contrast, genes downregulated upon tazemetostat treatment were enriched in GO terms related to angiogenesis, wound healing, and amino acid metabolism, while genes downregulated upon EZH2 knockdown were mostly associated with the cell cycle (Fig. 2E and Supplementary Fig. 2E). A heatmap visualizes the expression profiles of representative genes of each GO category (Fig. 2F). TRRUST analysis of DEGs predicted the involvement of transcription factors MYC, C/EBP, and HNF4A in upregulated genes, while NFκB, TP53, and STAT3 were predicted to regulate downregulated genes (Supplementary Fig. 2G). Taken together, our data suggest that EZH2 inhibition leads to a decrease in H3K27me3 levels, thereby reshaping gene expression profiles in HCC. Notably, the impact of EZH2 inhibition is manifest across pathways related to sulfur amino acid and lipid metabolism, wound healing, angiogenesis, and oxidative stress response, highlighting the integral role of EZH2-mediated H3K27me3 in orchestrating diverse biological processes in HCC.

### EZH2-mediated H3K27me3 regulates SAA metabolic gene expression in HCC

**Fig. 3:**
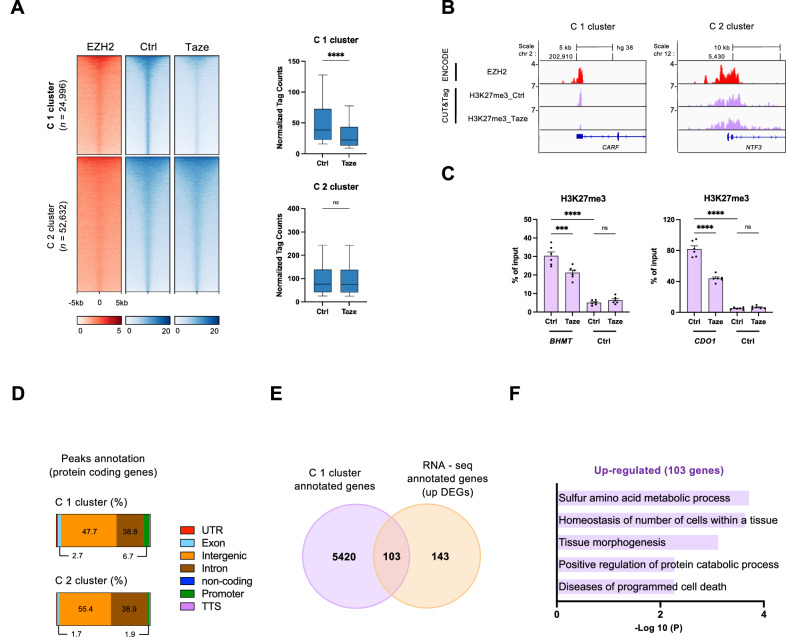
EZH2-mediated H3K27me3 regulates SAA metabolic processes in HCC. **A** Heatmap showing decreased H3K27me3 regions defined by CUT&Tag in two clusters (left panel). The bar plot shows the normalized tag counts in each cluster (right panel); ****p < 0.0001; unpaired *t*-test. **B** Representative IGV tracks displaying normalized tag density profiles at *CARF* and *NTF3*. **C** H3K27me3 levels at the promoter region of indicated genes were measured by CUT&Tag-qPCR analysis performed on HepG2 cells. Ctrl represents an intergenic region of chromosome 5 without H3K27me3 and serves as a negative control; ***p < 0.001, ****p < 0.0001; Ordinary one-way ANOVA. **D** The genomic distribution of protein-coding genes from annotated peaks in each cluster was determined using HOMER. **E** Venn diagram showing the number of overlapping genes between C1 cluster and the upregulated DEGs from RNA-seq. **F** GO analysis was performed with the intersected 103 genes using Metascape.

To unravel the intricate mechanisms governing the gene expression program influenced by EZH2-mediated histone modifications in HCC, we performed CUT&Tag experiments to map H3K27me3 in the HepG2 cell line under conditions of EZH2 inhibition using tazemetostat, as well as EZH2 knockdown. Our CUT&Tag data were validated by comparison with ENCODE H3K27me3 ChIP-seq data, revealing a significant overlap of 70% (68,182 peaks), also identifying an additional 143,228 peaks (Supplementary Fig. 3A, B). We identified two distinct clusters of genomic regions (C1 and C2) differentially regulated by EZH2 inhibition. The C1 cluster is characterized by chromatin regions where enriched H3K27me3 in the control was reduced upon EZH2 inhibition (Fig. 3A). In contrast, the C2 cluster displayed H3K27me3 levels that were not changed by EZH2 inhibition, suggesting regions insensitive to the EZH2 inhibitor (Fig. 3A). We confirmed the enrichment of H3K27me3 and EZH2 at the promoters of *CARF* and *NTF3* through our CUT&Tag and ENCODE ChIP-seq datasets (Fig. 3B). Upon treatment with tazemetostat, H3K27me3 levels were significantly reduced at the promoters of genes in the C1 cluster, such as *BHMT* and *CDO1* (Fig. 3A–C). Consistently, reductions in H3K27me3 levels were observed under EZH2 knockdown (Supplementary Fig. 3C–E). The peaks within each cluster (C1 and C2) were found to occupy varying proportions of regions, including intergenic, intronic, and promoter regions (Fig. 3D).

To delineate a core set of genes suppressed by EZH2-mediated transcriptional repression, we intersected the genes associated with the C1 cluster, enriched for H3K27me3, with the upregulated genes from our RNA-seq analysis upon EZH2 depletion (Fig. 2D), revealing 103 candidate genes (Fig. 3E). A subsequent GO analysis performed on these 103 genes uncovered potential biological functions, including sulfur amino acid and protein metabolic process, that are modulated by EZH2-H3K27me3-mediated gene expression (Fig. 3F). Notably, we detected a consistent alignment with the SAA metabolism process, as illustrated in Fig. 2E. These findings suggest that EZH2-driven H3K27me3 predominantly silences genes essential to certain metabolic pathways, exerting influence through epigenetic modifications in HCC.

### Enhanced chromatin accessibility upon EZH2 inhibition leads to gene derepression

**Fig. 4:**
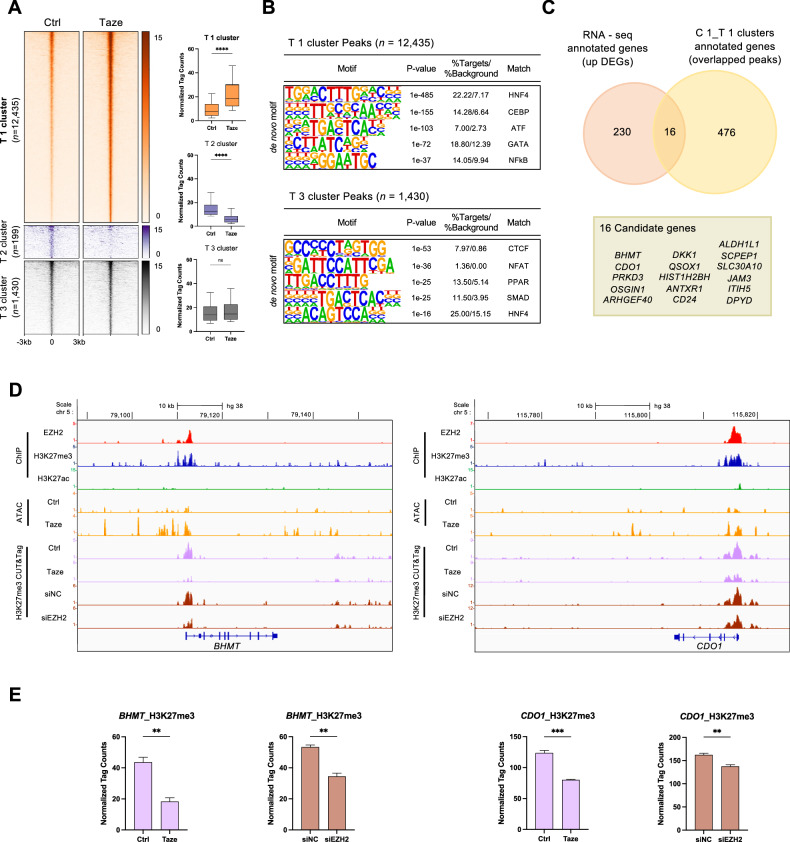
EZH2 inhibition leads to derepression of *BHMT* and *CDO1* through increased chromatin accessibility. **A** Heatmap showing distal accessible chromatin regions defined by ATAC-seq in three clusters (left panel). The bar plot shows the normalized tag counts in each cluster (right panel); ****p < 0.0001; unpaired *t*-test. **B** De novo motif analysis using HOMER identified the most significantly enriched transcription factor motifs. **C** Venn diagram showing 16 candidate genes that were revealed by intersections between DEGs in the following two categories: RNA-seq and C1_T1 overlapped cluster from CUT&Tag and ATAC-seq. **D** Representative IGV tracks showing normalized tag density and RNA expression levels in *BHMT* and *CDO1*. **E** The normalized tag counts for H3K27me3 of both *BHMT* and *CDO1* genes.

To unravel the chromatin alterations that enable gene derepression following EZH2 inhibition, we conducted ATAC-seq, identifying three distinct clusters. Strikingly, the T1 cluster (n = 12,435) displayed pronounced augmentation in chromatin accessibility upon tazemetostat treatment (Fig. 4A). Motif analysis revealed a cluster-specific enrichment of TF binding sites (Fig. 4B, upper panels). Within the T1 cluster, there was a notable enrichment of HNF4 motifs (*p* = 1 ×10^-485^), a known tumor suppressor involved in hepatocyte differentiation, along with CEBP (*p* = 1 × 10^−155^) and ATF (*p* = 1 × 10^−103^) motifs. Conversely, the T3 cluster was enriched in CTCF, NFAT, PPAR, SMAD, and HNF4 binding motifs (Fig. 4B, lower panels). The T2 cluster exhibited a lack of transcription factor motif enrichment, likely due to the limited number of peaks within this cluster. Remarkably, the peak distribution across clusters varied, with allocations across different regulatory domains such as intergenic, introns, and promoters. Specifically, peaks of the T1 cluster were predominantly situated within promoter regions compared to those in the T2 cluster (Supplementary Fig. 4A). We observed a significant overlap between genomic regions that gained chromatin accessibility (T1 cluster in ATAC-seq) and regions exhibiting decreased EZH2-mediated H3K27me3 (C1 cluster in CUT&Tag) upon tazemetostat treatment (Supplementary Fig. 4B). Motif enrichment analysis revealed that this C1_T1 overlapped cluster was enriched in binding motifs for transcription factors such as GATA, NFκB, HNF4, ATF, and CEBP (Supplementary Fig. 4C), suggesting their potential regulatory roles at these EZH2-modulated chromatin regions.

To identify candidate genes regulated by EZH2, we performed an integrative analysis by intersecting the upregulated DEGs from RNA-seq data upon EZH2 inhibition with genes that displayed decreased H3K27me3 levels and increased chromatin accessibility following EZH2 blockade (C1_T1 overlapped cluster from CUT&Tag and ATAC-seq data). Our integrative approach identified 16 candidate genes as potential EZH2 targets (Fig. 4C), with a particular focus on *BHMT* and *CDO1*, both of which are regulated by HNF4α and play crucial roles in directing sulfur amino acid metabolism. These findings are consistent with the GO enrichment results from the RNA-seq and CUT&Tag datasets (Figs. 2E and 3F). The IGV tracks illustrate that both *BHMT* and *CDO1*, initially repressed by EZH2-mediated H3K27me3, underwent derepression due to reduced H3K27me3 and enhanced chromatin accessibility following tazemetostat treatment (Fig. 4D). The normalized tag counts for H3K27me3 at both *BHMT* and *CDO1* gene loci were significantly decreased upon tazemetostat treatment and EZH2 knockdown (Fig. 4E). With the underlying hypothesis that inhibiting EZH2 could restore normal liver tissue characteristics, we examined HNF4α binding to the promoters or downstream enhancers of *BHMT* and *CDO1* using ENCODE HNF4α ChIP-seq datasets from normal liver tissue. The analysis confirmed HNF4α binding at these gene loci (Supplementary Fig. 4D). Consistent with the motif analysis, the normalized tag counts for HNF4α were significantly higher in the open chromatin regions compared to the closed ones (Fig. 4B and Supplementary Fig. 4E). Collectively, these data suggest that genes involved in cysteine metabolism, particularly *BHMT* and *CDO1*, are regulated by EZH2-mediated histone methylation, making them promising candidates for further investigation.

### EZH2-mediated suppression of *BHMT* and *CDO1* in HCC correlates with poor prognosis

**Fig. 5:**
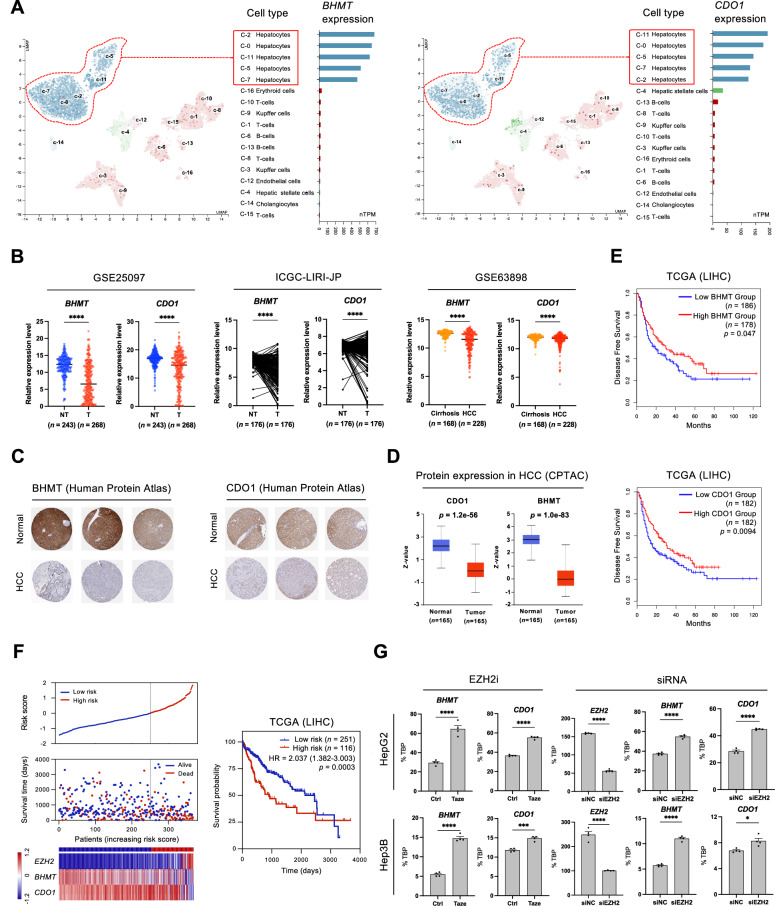
*BHMT* and *CDO1* are expressed in hepatocytes and suppressed in HCC, leading to poor prognosis. **A** Single-cell RNA-seq data analysis shows that *BHMT* and *CDO1* are expressed specifically in hepatocytes compared to other cell types in hepatic tissues. **B** *BHMT* and *CDO1* were suppressed in HCC tumors compared to non-tumor or cirrhosis tissues in three independent cohorts. **C**, **D** BHMT and CDO1 protein levels were decreased in tumor tissues compared to normal liver tissues, according to data from the HPA and CPTAC databases. **E** Kaplan–Meier survival plots show disease-free survival with high versus low expression of *BHMT* and *CDO1*. Patients were divided into high (50%) or low (50%) expression groups, except for disease-free survival based on *BHMT* expression, where patients were divided into high (51%) or low (49%) groups. **F** Risk score distribution, survival status profile, and a heatmap of gene expression levels in both high- and low-risk groups. The Kaplan-Meier analysis demonstrates that the survival probabilities of HCC patients were significantly lower in the high-risk group than in the low-risk group (*p* = 0.0003). **G** RT-qPCR results demonstrate derepression of *BHMT* and *CDO1* by EZH2 inhibition and specific inhibition of *EZH2* expression by siRNA transfection (*n* = 3). *TBP* was used for the normalization of each cell line level. *P* values were determined by unpaired *t*-test (*p < 0.05, **p < 0.01, ***p < 0.001, ****p < 0.0001).

To determine the specific cell populations in healthy liver tissue that express *BHMT* and *CDO1* genes, we analyzed scRNA-seq data from 8439 cells, stratified into 17 unique clusters, obtained from liver samples of five healthy individuals featured in the Human Protein Atlas (HPA) dataset. Both *BHMT* and *CDO1* were robustly expressed in healthy liver, primarily in hepatocytes relative to other cell populations (Fig. 5A). We corroborated the expression levels of *BHMT* and *CDO1* in various HCC cohorts and observed a marked suppression in HCC tumors compared to normal and cirrhosis samples across all independent cohorts examined (Fig. 5B and Supplementary Fig. 5A). Moreover, protein levels of BHMT and CDO1 were markedly reduced in HCC patients (Fig. 5C, D). Notably, the patient group with reduced *BHMT* and *CDO1* expression showed significantly shorter overall survival and disease-free survival (Fig. 5E and Supplementary Fig. 5B). To further investigate the correlation between *EZH2, BHMT*, and *CDO1* in TCGA LIHC cohort, we performed univariate Cox analysis to develop a risk signature. We first calculated the risk score for each LIHC patient by summing these three gene scores to define the high-risk group. Each gene’s score was derived by multiplying the log_2_-transformed gene expression value (FPKM + 1) by its corresponding coefficient from the Cox regression. Then, HCC patients were classified into high- and low-risk groups based on a zero cut-off value. The high-risk group (116 patients) exhibited higher *EZH2* expression, while the low-risk group (251 patients) displayed elevated *BHMT* and *CDO1* levels. Kaplan-Meier analysis revealed a significantly worse prognosis (p = 0.0003) for the high-risk group, characterized by high *EZH2* expression and low *BHMT* and *CDO1* expression (Fig. 5F).

To elucidate the influence of EZH2 inhibition on BHMT and CDO1, we employed two distinct experimental approaches. Initial assays with tazemetostat treatment revealed a pronounced increase in the expression of *BHMT* and *CDO1* (Fig. 5G, left panels). Subsequently, we utilized siRNA-mediated knockdown of EZH2 to effectively diminish its expression in HCC cell lines. This intervention resulted in a marked reduction of *EZH2* mRNA levels and a concomitant increase in *BHMT* and *CDO1* expression (Fig. 5G, right panels), consistent with the effects of functional inhibition of EZH2. Furthermore, we observed that EZH2 suppresses *HNF4a* expression in the HNF4α-negative cell line (SNU-449), while EZH2 inhibition substantially upregulated *BHMT*, *CDO1*, and *HNF4a* in this cell line (Supplementary Fig. 5C, D). Collectively, these findings demonstrate that *BHMT* and *CDO1* are highly expressed in normal hepatocytes but significantly suppressed in HCC cells. The reduction in their expression correlates with unfavorable survival rates in HCC patients, emphasizing the potential therapeutic role of EZH2 inhibition in restoring their expression.

### EZH2 inhibition augments lipid ROS through cysteine metabolism and ferroptosis suppressors in HCC

**Fig. 6:**
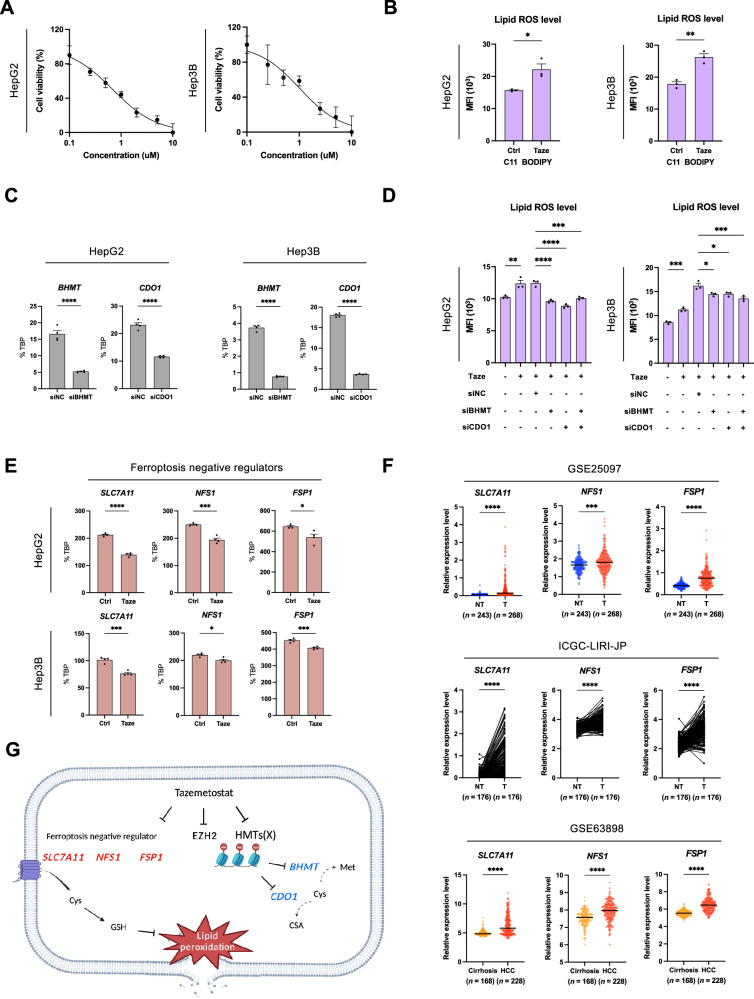
EZH2 inhibition increases lipid ROS by regulating cysteine metabolism-related genes and ferroptosis repressors. **A** Cell viability (% normalized to control) of HepG2 and Hep3B cells treated with increasing concentrations of tazemetostat (*n* = 3). **B** Lipid peroxidation levels in HepG2 and Hep3B cells treated with DMSO or tazemetostat (5 μM) for 5 days (*n* = 3). **C** RT-qPCR analysis of *BHMT* and *CDO1* expression following siRNA-mediated knockdown of *BHMT* or *CDO1* (*n* = 3). **D** Lipid peroxidation levels in HepG2 and Hep3B cells following siRNA-mediated knockdown of *BHMT* or *CDO1*, with or without tazemetostat treatment for 5 days (*n* = 3). **E** RT-qPCR analysis of ferroptosis negative regulators *SLC7A11*, *NFS1*, and *FSP1* expression following tazemetostat treatment (*n* = 3). **F** Expression of *SLC7A11*, *NFS1*, and *FSP1* in HCC tumors versus non-tumor or cirrhotic tissues across three independent cohorts. **G** Schematic of EZH2 inhibition effects on gene expression program in HCC. *TBP* was used for the normalization of each cell line level. *P* values were determined by unpaired *t*-test (*p < 0.05, **p < 0.01, ***p < 0.001, ****p < 0.0001).

Given the roles of BHMT and CDO1 in cysteine homeostasis and the established link between cysteine depletion and ferroptosis, we investigated the impact of EZH2 inhibition on intracellular cysteine and lipid ROS levels in HCC. Treatment with the EZH2 inhibitor tazemetostat resulted in dose-dependent reductions in cell viability and concomitant increases in lipid ROS in HepG2 and Hep3B cell lines (Fig. 6A, B). Knockdown of *BHMT* and *CDO1* attenuated both basal and tazemetostat-induced lipid ROS accumulation (Fig. 6C, D and supplementary Fig. 6A). However, this knockdown did not restore cell viability (Supplementary Fig. 6B), suggesting the involvement of additional mechanisms in EZH2 inhibition-mediated cell death.

To further elucidate the mechanisms underlying EZH2 inhibition-induced ferroptosis, we examined other ferroptosis-related genes regulated by EZH2. Notably, EZH2 inhibition downregulated *SLC7A11*, a cysteine uptake transporter and negative regulator of ferroptosis (Figs. 2D and 6E). Expression of additional ferroptosis suppressors, *NFS1* and *FSP1* [51–54], was also reduced following EZH2 inhibition (Fig. 6E and Supplementary Fig. 6C). These findings collectively suggest that EZH2 inhibition enhances ferroptosis through combined effects on cysteine metabolism genes and key ferroptosis suppressors in HCC. To assess the clinical relevance of ferroptosis-negative regulators in HCC, we analyzed four independent HCC patient cohorts. This analysis revealed elevated expression of *SLC7A11, NFS1*, and *FSP1* in HCC compared to normal and cirrhotic tissues (Fig. 6F and Supplementary Fig. 6D). Collectively, our results indicate that EZH2 inhibition may enhance lipid ROS accumulation by epigenetically modulating genes associated with cysteine metabolism and ferroptosis suppression in HCC (Fig. 6G).

## Discussion

EZH2, frequently overexpressed in various cancers including HCC, has been investigated to mediate tumor suppression upon its inhibition through diverse pathways [12–15, 20]. Recent studies have further highlighted the potential of targeting EZH2 to refine therapeutic strategies for liver cancer [55]. However, the precise mechanisms by which EZH2 inhibition confers therapeutic benefits remain to be fully elucidated. In this study, by integrating insights from both transcriptomic and epigenomic analyses, we shed light on the functional implications of genes affected by EZH2 inhibition in HCC. Of significance, we identified marked alterations in genes linked to cysteine metabolism, hinting at the modulatory role of EZH2 inhibition on this metabolic pathway.

Our comprehensive epigenomic approach, integrating H3K27me3 CUT&Tag and ATAC-seq data following treatment with the EZH2 inhibitor tazemetostat, revealed chromatin regions exhibiting diminished EZH2-mediated H3K27me3 levels concomitant with enhanced chromatin accessibility. Intriguingly, motif analysis of these chromatin regions regulated by EZH2 inhibition indicated an overrepresentation of the transcription factor HNF4α. Depletion of HNF4α has been linked to abnormalities in SAA metabolism, inclusive of *BHMT* and *CDO1*, culminating in methionine restriction, sorafenib resistance, and epithelial-mesenchymal transition [37]. Our findings suggest that EZH2 inhibition potentially derepresses *BHMT* and *CDO1* expression, with HNF4α binding to newly accessible chromatin regions to activate these genes. In the HNF4α-negative HCC cell line treated with tazemetostat, we observed a marked upregulation of *HNF4a, BHMT*, and *CDO1*, suggesting that EZH2 exerts a suppressive effect on HNF4α expression. Furthermore, downregulation of *SLC7A11*, a key transporter for cystine uptake, can trigger ferroptosis through cysteine deprivation [39]. During tazemetostat treatment, the upregulation of the *BHMT* and *CDO1* genes may potentially limit the intracellular cysteine availability *via* the transsulfuration pathway. Concurrently, decreased *SLC7A11* expression suggests an impediment in external cysteine acquisition. This suggests a potential induction of ferroptosis, excluding lipid ROS inhibition due to the suppressed cysteine-glutathione-glutathione peroxidase 4 (cys-GSH-GPX4) cascade.

Our findings reveal that while *BHMT* and *CDO1* knockdown reduced lipid ROS levels, this reduction alone was insufficient to restore cell viability. Recent evidence suggests that siRNA-mediated gene silencing may sensitize cells to ferroptosis through off-target effects, potentially via depletion of intracellular glutathione and concomitant upregulation of GPX4 [56]. Our observations corroborate this, as treatment with non-targeting control siRNA increased basal lipid ROS levels and cell death. These findings underscore the potential limitations of siRNA-based approaches in precisely elucidating the effects of target gene downregulation on ferroptosis. Furthermore, the complex molecular landscape governing ferroptosis suggests that modulation of individual genes may be insufficient to comprehensively control this cell death pathway. As highlighted by Nakamura and Conrad [57], simultaneous targeting of multiple ferroptosis-related pathways, such as the GPX4 and FSP1 axes, may provide a more potent strategy for ferroptosis induction in cancer cells, particularly in therapy-resistant malignancies. Taken together, our results indicate that EZH2 inhibition may induce ferroptosis in HCC through the combinatorial effects of key cysteine metabolism genes and other negative regulators of ferroptosis.

Sorafenib, a standard first-line therapeutic agent for HCC, was originally pinpointed for its inhibitory role against Raf/serine/threonine kinase. However, its broader implications have become evident, including the induction of ferroptosis *via* SLC7A11 inhibition, stimulation of apoptosis, attenuation of angiogenesis, and inhibition of tumor cell proliferation [58–61]. Furthermore, epigenetic modulation can alter the tumor microenvironment and regulate drug resistance, including resistance to sorafenib, by controlling gene expression without altering DNA sequences [61]. Notably, H3K27me3 levels have been reported to increase with sorafenib treatment and decrease with co-administration of an EZH2 inhibitor in HCC [15]. Given these insights, combination therapy is increasingly utilized, and our study adds to the growing evidence that targeting overexpressed EZH2 in liver cancer might prove effective in conjunction with other therapeutic modalities [62]. Specifically, our findings propose a novel therapeutic direction where EZH2 inhibition regulates cysteine metabolism and potentially mitigates sorafenib resistance, thus enhancing the induction of ferroptosis in HCC.

Although our study did not directly investigate whether EZH2 regulates non-histone proteins to activate downstream genes independently of the PRC2, these non-canonical functions of EZH2 may contribute to the downregulation of genes observed upon EZH2 inhibition. For example, AKT-induced phosphorylation of EZH2 at Ser21 can lead to the methylation of STAT3, promoting its activation [63]. Furthermore, EZH2 has been shown to interact with NFκB, resulting in the activation of genes associated with triple-negative breast cancer [64]. Our transcription factor (TF) prediction analysis of downregulated genes from RNA-seq data upon EZH2 inhibition identified NFκB and STAT3 as potential targets. This suggests that tazemetostat, may suppress gene expression by regulating these transcription factors in an H3K27me3-independent manner [65]. Additionally, the transactivation domain of EZH2 has been demonstrated to bind directly with androgen receptor and its variant, functioning as a co-activator to transcribe oncogenes, and EZH2 can interact with the transcriptional co-activator and histone acetyltransferase p300, thereby leading to the activation of gene expression in a p300-dependent manner [66, 67]. The downregulation of ferroptosis negative regulators such as *SLC7A11, NFS1*, and *FSP1* upon EZH2 inhibition could potentially be attributed to these different mechanisms. In tongue squamous cell carcinoma, EZH2 repression of miR-125b-5p has been reported, leading to enhanced SLC7A11 expression, thereby averting erastin-induced ferroptosis [68]. This spectrum of findings underscores the potential avenues for future investigations into EZH2-mediated gene regulation, focusing on non-histone target mechanisms.

In summary, our study provides clarity on the multifarious mechanisms by which EZH2 inhibition influences a spectrum of gene sets governing diverse functions such as cell death, spanning both epigenomic and transcriptomic levels. Such insights are pivotal for comprehending the intricacies of gene regulation in the context of EZH2 inhibition and its concomitant pathological implications. Thus, our study sets the stage for a more detailed examination of therapeutic approaches targeting EZH2 in the context of HCC management.

## Materials and methods

### Transcriptomic data analysis

The overall survival (OS) and disease-free survival (DFS) of patients based on the expression levels of *EZH2*, *SUZ12*, *EED*, *BHMT*, and *CDO1* in liver hepatocellular carcinoma (LIHC), kidney renal clear cell carcinoma (KIRC), breast invasive carcinoma (BRCA), colon adenocarcinoma (COAD), lung squamous cell carcinoma (LUSC), and stomach adenocarcinoma (STAD) were determined using the Gene Expression Profiling Interactive Analysis 2 (GEPIA2) tool, which utilizes data from The Cancer Genome Atlas (TCGA) database [69]. Expression profile data of GSE14520 (GPL_3921 subset 220 nontumor, 225 HCC samples), GSE25097 (243 nontumor, 268 HCC samples), GSE63898 (168 cirrhosis and 228 HCC samples), and international cancer genome consortium liver cancer RIKEN Japan (ICGC-LIRI-JP; 176 nontumor; 176 HCC samples) were also downloaded from gene expression omnibus (GEO, https://www.ncbi.nlm.nih.gov/geo/) and HCCDB (http://lifeome.net/database/hccdb/home.html), respectively [70–72]. These independent cohort data were used as a test set for external validation. Immunohistochemical (IHC) protein expression data for BHMT, and CDO1 in normal and cancerous liver tissues were downloaded from the Human Protein Atlas (HPA) database (https://www.proteinatlas.org/). Protein expression levels for EZH2, SUZ12, EED, BHMT, and CDO1 in normal and tumor were determined using Clinical Proteomic Tumor Analysis Consortium (CPTAC) database. To identify cell types expressing *BHMT* and *CDO1* in hepatic tissues, scRNA-seq data analysis was conducted with the HPA database.

### Gene Ontology analysis

Gene ontology (GO) analysis was performed using Metascape (http://metascape.org/) [73] with default parameters. For the RNA-seq data, significantly upregulated or downregulated genes were analyzed to gain insights into their functional implications. For the CUT&Tag data, peak-associated genes from the C1 cluster were overlapped with RNA-seq data and used as input for the analysis.

### Data visualization

Heatmaps of CUT&Tag, ChIP-seq, and ATAC-seq data were generated using deepTools (https://deeptools.readthedocs.io/) [74] to visualize signal densities across gene-associated regions or genomic loci. Snapshots of specific genomic loci were obtained using the Integrated Genome Viewer (IGV) [75].

### Cell culture

The human liver cancer cell lines HepG2, Hep3B, and SNU-449 were obtained from the Korean Cell Line Bank (KCLB). HepG2 and Hep3B cells were cultured in Eagle’s minimum essential medium and dulbecco’s modified eagle medium (MEM; catalog no. 11095-080; DMEM; catalog no. 11965-092; Gibco, Grand Island, NY, USA). SNU-449 cell was cultured in RPMI-1640 medium (catalog no. 11875-093; Gibco). All media were supplemented with 10% fetal bovine serum (FBS; catalog no. 16000-044; Gibco) and 1% penicillin-streptomycin (catalog no. 15070-063; Gibco) or antibiotic-antimycotic (catalog no. 15240-062; Gibco). The cells were routinely cultured at 37 °C in a humidified incubator with 5% CO_2_.

### Western blot analysis

For the preparation of nuclear lysates, the cells were lysed with buffer A (10 mM HEPES pH 7.9, 10 mM KCl, 1.5 mM MgCl_2_, 340 mM Sucrose, 10% Glycerol, 0.1% Triton X-100, and protease inhibitors) and then centrifugated at 4500 rpm for 10 min at 4 °C. The nuclear pellet was washed with buffer A by centrifugation at 4500 rpm for 5 min at 4 °C and then lysed with SDS lysis buffer (20 mM Tris pH 7.5, 50 mM NaCl, 2.5% SDS, 2.5% Sodium deoxycholate, 0.5 mM PMSF, and protease inhibitors). The nuclear lysates were resolved by SDS-PAGE, followed by immunoblotting with the indicated antibodies: EZH2 (catalog no. 5246; Cell Signaling Technology), H3 (catalog no. ab1791; Abcam), H3K27me1 (catalog no. 07-448; Sigma-Aldrich), H3K27me2 (catalog no. 07-452; Sigma-Aldrich), H3K27me3 (catalog no. 07-449; Sigma-Aldrich) and α-Lamin A/C (catalog no. sc-376248; Santa Cruz Biotechnology).

### Assessment of H3K27me3 levels using flow cytometry

HepG2 and Hep3B cells, approximately 200,000 cells per sample, were treated with tazemetostat (catalog no. HY-13803; MedChemExpress, Monmouth Junction, NJ, USA). The cells were then harvested using TrypLE Express (catalog no. 12604013; Gibco). To detect intracellular H3K27me3 levels, the cells were fixed and permeabilized at room temperature for 20 min using Cyto-Fast^TM^ Fix/Perm buffer (catalog no. 426803; BioLegend, San Diego, CA, USA). After fixation and permeabilization, the cells were washed twice with 1X Cyto-Fast^TM^ perm wash buffer and incubated with anti-H3K27me3 antibody (catalog no. 12158; Cell Signaling Technology, Danvers, MA, USA) diluted 1:50 in perm wash buffer for one hour at room temperature. Following antibody staining, the cells were washed with 1X Cyto-Fast^TM^ perm wash buffer and cell-staining buffer (catalog no. 420201; BioLegend). The stained cells were then analyzed using a CytoFLEX flow cytometer (Beckman Coulter, Brea, CA, USA), and the levels of H3K27me3 were quantified using FlowJo v10.7 software (FlowJo LLC, Ashland, CO, USA).

### RNA extraction and real-time quantitative polymerase chain reaction

Total RNA was extracted from cells using the Ribospin II kit (GeneAll Biotechnology, Seoul, Korea). Reverse transcription of 0.5 μg of total RNA was performed using the RevertAid First Strand cDNA Synthesis kit (catalog no. K1622; Thermo Fisher Scientific, Waltham, MA, USA). Real-time quantitative polymerase chain reaction (qPCR) was performed using the TOPreal qPCR 2X PreMIX (SYBR Green with low ROX; No. RT500M; Enzynomics Co. Ltd., Daejeon, Korea). The real-time qPCR conditions were as follows: one cycle at 95 °C for 10 min, followed by 50 cycles of denaturation at 95 °C for 10 s, annealing at 60 °C for 15 s, and extension at 72 °C for 20 s. A melting program was performed at 72 °C to 95 °C with a heating rate of 1 °C/45 s. The spectral data were captured and analyzed using the Rotor-Gene Q v2.3.1 instrument (Qiagen, Hilden, Germany).

### RNA-seq analysis

After RNA extraction, libraries for sequencing were prepared using the Illumina TruSeq Stranded Total RNA Library Prep Kit, according to the manufacturer’s instructions. High-throughput paired-end sequencing was performed using an Illumina NovaSeq 6000 Sequencer at Theragen (Seongnam, Korea), resulting in an average of 28 million reads per sample. The sequenced reads were then mapped to the reference human genome (hg38 assembly) using the STAR aligner with default parameters [76]. The expression levels of genes in each sample were normalized by means of transcript per million (TPM). Differentially expressed genes (DEGs) were identified using edgeR with raw read counts. To further obtain reliable DEGs, the getDifferentialExpression command from HOMER was used with the following thresholds: adjusted *p*-values < 0.05, 1.3-fold differences in expression, and TPM > 1.5.

### Omni-Assay for Transposase-Accessible Chromatin (ATAC) protocol

Tazemetostat (EPZ-6438)-treated HepG2 cells were pretreated with 200 U/ml DeoxyriboNuclease I (catalog no. LS002004, Worthington Biochem, Lakewood, NJ, USA) for 30 min at 37 °C to remove free-floating DNA and digest DNA from dead cells. The cells were then washed, resuspended in cold phosphate-buffered saline (PBS), and counted. 50,000 cells were resuspended in 1 mL of cold ATAC-seq resuspension buffer (RSB; 10 mM Tris-HCl pH 7.5 (catalog no. 15567-027; Invitrogen, Carlsbad, CA, USA), 10 mM NaCl (catalog no. AM9759; Invitrogen), and 3 mM MgCl2 (catalog no. AM9530G; Invitrogen) in nuclease-free water) and centrifuged at 500 relative centrifugal force (RCF) for 5 min in a pre-chilled (4 °C) fixed-angle centrifuge. After centrifugation, the supernatant was carefully aspirated to avoid the cell pellet, and the cells were then resuspended in 50 μl of RSB containing 0.1% NP40 (catalog no. 11332473001; Roche, Penzberg, Upper Bavaria, Germany), 0.1% Tween-20 (catalog no. 11332465001; Roche), and 0.01% digitonin (catalog no. G9441; Promega, Madison, WI, USA). This cell lysis reaction was incubated on ice for 3 min. After lysis, 1 ml of RSB containing 0.1% Tween-20 (without NP40 or digitonin) was added, and the tubes were inverted to mix. Nuclei were then centrifuged for 10 min at 500 RCF in a pre-chilled (4 °C) fixed-angle centrifuge. The supernatant was removed, and nuclei were resuspended in 50 μl of transposition mix (25 μl TD Tagment DNA Buffer (catalog no. 15027866; Illumina, San Diego, CA, USA), 2.5 μl TDE1 Tagment DNA Enzyme (catalog no. 15027865; Illumina), 16.5 μl DPBS, 0.5 μl 1% digitonin, 0.5 μl 10% Tween-20, and 5 μl Nuclease-Free Water). The transposition reactions were incubated at 37 °C for 30 min in a thermomixer with shaking at 1000 revolutions per minute (RPM). Reactions were cleaned up with MinElute Reaction Cleanup Kit (catalog no. 28206, Qiagen) and pre-amplified for 5 cycles using NEBNext High-Fidelity 2x PCR MasterMix (catalog no. M0541L; NEB, Ipswich, MA, USA) with adapter primers. The information of adapter primer sequences is consulted from Buenrostro et al. [77]. The amplification profiles were manually assessed to determine the number of additional cycles to amplify as previously described [78]. The final amplified library samples were stored at -80 °C for ATAC-seq after library purification with AMPure XP (catalog no. A63880; Beckman Coulter). Libraries were validated using the Agilent High Sensitivity D1000 kit (catalog no. 5067-5584, 5067-5585; Agilent, Santa Clara, CA, USA) and were subjected to high-throughput sequencing using the Illumina HiSeq 2500 Sequencer.

### Cleavage Under Targets and Tagmentation (CUT&Tag)

CUT&Tag was performed using the single-tube protocol (Option B) as described by Kaya-Okur et al. [79]. Briefly, HepG2 cells were washed with PBS and resuspended in NE1 buffer (20 mM HEPES-KOH pH 7.9, 10 mM KCl, 0.5 mM Spermidine, 0.1% Triton X-100, 20% Glycerol, 1x protease inhibitors). After incubation on ice for 10 min, nuclei were centrifuged and washed once with PBS. Nuclei were then fixed with 0.1% formaldehyde for 2 min at room temperature, and the reaction was quenched with glycine. Fixed nuclei were resuspended in wash buffer (20 mM HEPES pH 7.5, 150 mM NaCl, 0.5 mM Spermidine, 1x protease inhibitors) and counted. 1 × 10^^^5 nuclei were used per CUT&Tag reaction. Nuclei were bound to activated concanavalin A-coated magnetic beads (catalog no. 21-1401; Epicypher) and incubated with an H3K27me3 antibody (catalog no. C36B11; Cell Signaling Technology) in antibody buffer overnight at 4°C. After washing in wash buffer, samples were incubated with a goat anti-rabbit secondary antibody (catalog no. ab182016; Abcam) in wash buffer for 30 min at room temperature, followed by an additional wash. After further wash in 300-wash buffer (20 mM HEPES pH 7.5, 300 mM NaCl, 0.5 mM Spermidine, 1x protease inhibitors), samples were incubated with pAG-Tn5 (catalog no. 15-1017; Epicypher) for 1 h at room temperature. After additional washes, tagmentation was carried out in 300-wash buffer containing 10 mM MgCl2 for 1 h at 37°C. To release pA-Tn5 and cleaved chromatin fragments, samples were resuspended in 0.1% SDS release solution, incubated at 58°C for 1 h, and neutralized by the addition of 0.67% Triton-X100. PCR was performed by directly adding NEBNext HiFi PCR Master Mix (catalog no. M0541L; NEB) and sample-specific barcoded adapters to the tagmented fragments. After amplification, libraries were cleaned up using AMPure beads (catalog no. A63880; Beckman Coulter). The prepared libraries were sequenced using 150-base pair paired-end sequencing on an Illumina Novaseq platform.

### CUT&Tag, ChIP-seq, and ATAC-seq data analysis

The raw files (FASTQ) of EZH2, H3K27me3, and H3K27ac ChIP-seq in HepG2 cell line (GSE29611) and HNF4α in normal liver tissue (GSE96260) were downloaded from the ENCODE dataset (https://www.encodeproject. org/) [80]. The raw files for CUT&Tag and ATAC-seq were generated as part of this study. To ensure data quality, the raw files were trimmed using Trim Galore (version 0.6.6, https://www.bioinformatics.babraham.ac.uk/projects/trim_galore/) with default parameters. These trimmed reads were then aligned to the human genome (hg38 assembly) using Bowtie2 (version 2.3.5.1) with the following parameters: --end-to-end --very-sensitive --no-unal --no-mixed --no-discordant --phred33 -I 10 -X 700 for CUT&Tag, with --very-sensitive --no-discordant -X 2000 for ATAC-seq, and with default parameters for ChIP-seq [81]. To identify peaks over background signals, the makeTagDirectory and findPeaks commands from HOMER (version 4.11.1) [82] were utilized. For CUT&Tag and ATAC-seq data analysis, the FDR cutoff of 0.05 was employed. The total number of mapped reads for each sample was normalized to 10 million mapped reads. Differential peak analysis for the CUT&Tag and ATAC-seq data was performed using the getDifferentialPeaks command. Overlapping peaks were obtained by intersecting differential peaks, which were then used for further analysis.

### Motif enrichment analysis

To identify enriched motifs representing transcription factor binding sites, a de novo motif analysis was performed with ATAC-seq peaks using the findMotifsGenome.pl function from the HOMER package.

### Construction and assessment of risk score system

For the development of the risk score, we utilized patient data from the TCGA-LIHC, obtained from the UCSC Xena platform. The prognostic value of each gene was first assessed using univariate Cox proportional hazards regression. The risk score for each LIHC patient was calculated as the sum of individual gene scores. Each gene’s score was derived by multiplying the log2-transformed gene expression value (FPKM + 1) by its corresponding coefficient derived from the Cox regression. Using zero as the cut-off value for the risk score, we divided HCC patients into high- and low-risk groups. The risk score model of each patient was calculated using the following formula:

$${risk\; score}=\mathop{\sum }\limits_{i}^{n}{{coe}}_{i}* {\exp }_{i}$$

In the formula used for risk score calculation, coe_i_ represents the univariate coefficient for each gene_i_, while exp_i_ is the expression value of each gene_i_. The summation is performed over n, the total number of tested genes, which in this case is 3. Scatter plot and Kaplan-Meier curve was produced by the GraphPad Prism v. 9 (GraphPad Software, La Jolla, CA, USA) using the risk score. Comparison of two survival curves were conducted using the Log-rank (Mantel-Cox) test.

### RNA interference

The *EZH2*, *BHMT,* and *CDO1* genes were knocked down with SMART pool ON‐TARGETplus siRNA (catalog no. L‐015700‐00‐0005, L-008422-00-0005, L-010336-00-0005; Dharmacon Inc., Lafayette, CO, USA). The ON‐TARGETplus Non‐targeting Pool was used as a negative control (catalog no. D‐001810‐10‐05; Dharmacon Inc.). HepG2 and Hep3B cells were seeded into a six‐well plate and incubated for 24 hs. The cells were then transfected with the RNA duplex and Lipofectamine^TM^ RNAi‐MAX (catalog no. 13778075, Invitrogen) in Opti‐MEM I reduced serum medium (catalog no. 31985-062; Thermo Fisher Scientific) without antibiotics, according to the manufacturer’s instructions. After 24 hs, the medium was replaced with complete medium containing antibiotics, and the cells were harvested for subsequent experiments after an additional 24-hour incubation with siEZH2 and 72-hour incubation with siBHMT and siCDO1 at 37 °C in a humidified 5% CO_2_ incubator.

### CCK‐8 assay

Cell viability was measured using the CCK‐8 assay (catalog no. ab228554, Abcam, Cambridge, UK). HepG2 and Hep3B cells were seeded at a density of 1×10^4^ HepG2 cells and 5×10^3^ Hep3B cells per well in 96‐well microtiter culture plates and incubated overnight at 37 °C. The cells were then treated with specified concentrations of tazemetostat for 5 days. After the treatment, 10 μl of CCK‐8 solution was added to each well, followed by an additional 2-hour incubation. The absorbance of the samples was measured at 460 nm using an Epoch microplate spectrophotometer (BioTek Instruments, Winooski, VT, USA).

### Lipid peroxidation detection

Lipid ROS levels were assessed using the C11-BODIPY assay (catalog no. D3861, Invitrogen). Flow cytometry analysis was performed following published protocols [83]. HepG2 and Hep3B cells were incubated with 2 μM BODIPY581/592 c11 for 20 min at 37 °C, washed with DPBS and resuspended in DPBS for flow cytometric analysis. The cells were analyzed using a CytoFLEX instrument (Beckman Coulter).

### Statistical analysis

Statistical analysis was performed using GraphPad Prism v. 9, unless otherwise noted. Error bars represent the standard error of the mean (SEM), unless stated otherwise in the figure legend. Unpaired *t*-test and ordinary one-way ANOVA were used where appropriate. In all statistical analyses, groups that were compared had similar expected variances, and statistical significance was accepted at the 95% confidence level (*p < 0.05, **p < 0.01, ***p < 0.001, ****p < 0.0001).

## Supplementary information

Supplementary information

Supplementary Figures

Supplemental MateriaL

## Data Availability

All raw and processed data are deposited into the GEO database under accession number GSE237952.
