# Supplementary material for: Integration of epigenomic and transcriptomic profiling uncovers EZH2 target genes linked to cysteine metabolism in hepatocellular carcinoma
Source: Cell Death Dis. 2024 Nov 8;15(11):801. doi: 10.1038/s41419-024-07198-0 (PMC11549485; doi:10.1038/s41419-024-07198-0)
Supplement: Supplementary file 1 — Supplementary information [file 41419_2024_7198_MOESM1_ESM.docx]

**Integration of epigenomic and transcriptomic profiling uncovers EZH2 target genes linked to cysteine metabolism in hepatocellular carcinoma**

Jaehyun Lee^1^, Chaelin You^1^, Geunho Kwon^1^, Junho Noh^1^, Kyubin Lee^1^, Kyunghwan Kim^1^, Keunsoo Kang^2, *^, Kyuho Kang^1, *^

^1^ Department of Biological Sciences and Biotechnology, Chungbuk National University, Cheongju 28644, Korea

^2^ Department of Microbiology, College of Science & Technology, Dankook University, Cheonan 31116, Korea

^*^ Correspondence: [kangk@cbnu.ac.kr](mailto:kangk@cbnu.ac.kr); Tel.: +82-43-261-2295, [kangk1204@dankook.ac.kr](mailto:kangk1204@dankook.ac.kr); Tel.: +82-41-550-3456

**Supplementary Figure Legends**

**Supplementary Fig. 1**

*EZH2* is overexpressed in HCC compared to other cancers. **A** Kaplan-Meier survival plots showing overall survival with high vs low expression of *EZH2* in BRCA, COAD, LUSC, and STAD. Patients were divided into high (50%) or low (50%) expression groups. **B** Protein expression levels of EZH2, SUZ12 and EED were verified in KIRC tumors compared to non-tumor tissues in CPTAC database. **C** The RNA expression levels of *EZH2*, *SUZ12*, and *EED* were overexpressed in HCC tumors compared to non-tumor tissues.

**Supplementary Fig. 2**

EZH2 inhibition reverses gene expression program. **A** Experimental design: RNA-seq and CUT&Tag were performed on HepG2 cells after 48 hours of tazemetostat (5 μM) treatment, and following 24 hours of EZH2 knockdown with siRNA, while ATAC-seq was conducted after the same duration of tazemetostat treatment. **B** The RNA expression levels of *IGFBP4*, *CDH1*, and *PCK1* from the tazemetostat-treated RNA-seq data in HepG2. **C** The RNA expression levels of *EZH2* from the EZH2 knockdown RNA-seq data in HepG2. **D** Volcano plot of transcriptomic changes between siNC and EZH2 knockdown cells, with colored dots representing genes with significant (FDR < 0.05) and greater than 1.3-fold expression changes. **E** Biological pathways of DEGs in response to EZH2 knockdown using Metascape. **F** Venn diagram showing the number of overlapping genes between each set of up-regulated DEGs (right panel), or between each set of down-regulated DEGs (left panel) from RNA-seq with tazemetostat-treated and with EZH2 knockdown. GO analysis was performed with the intersected 39 or 54 genes using Metascape. **G** The transcription factor prediction of up- and downregulated transcripts upon tazemetostat treatment in HepG2 cells was analyzed by TRRUST. P values were determined by unpaired *t* -test (* p < 0.05, ** p < 0.01, *** p < 0.001, **** p < 0.0001).

**Supplementary Fig. 3**

Decreased histone methylation levels in H3K27me3 CUT&Tag following tazemetostat treatment and EZH2 knockdown. **A-B** The Venn diagram shows that H3K27me3 CUT&Tag peaks encompass H3K27me3 ChIP-seq peaks from ENCODE dataset. Representative IGV tracks displaying EZH2-H3K27me3 in the same promoter regions. **C** EZH2 knockdown heatmap showing decreased H3K27me3 regions defined by tazemetostat-treated CUT&Tag in two clusters (left panel). The bar plot shows the normalized tag counts in each cluster (right panel). **D** Representative IGV tracks displaying normalized tag density profiles at CARF and NTF3. **E** H3K27me3 levels at the promoter region of indicated genes were measured by CUT&Tag-qPCR analysis performed on HepG2 cells. Ctrl represents an intergenic region of chromosome 5 without H3K27me3 and serves as a negative control.; **** p < 0.0001; Ordinary one-way ANOVA.

**Supplementary Fig. 4**

*BHMT* and *CDO1* possess regions where HNF4α can bind under increased chromatin accessibility with EZH2 inhibition. **A** The genomic distribution of protein coding genes from annotated peaks in each cluster was determined using HOMER. **B** The heatmap displays intersected peaks from the C1 cluster of CUT&Tag and the T1 cluster of ATAC-seq, indicating regions suppressed by EZH2-H3K27me3 and opened with decreased H3K27me3 following tazemetostat treatment (left panel). The bar plot shows the normalized tag counts in each target (right panel) **C** De novo motif analysis conducted using HOMER, identifying the most significantly enriched transcription factor motifs. **D** Representative IGV tracks displaying normalized tag density profiles at *BHMT* and *CDO1*. **E** The bar plot shows the normalized tag counts of HNF4α peaks in each cluster of ATAC-seq. P values were determined by ordinary one-way ANOVA ( **** p < 0.0001).

**Supplementary Fig. 5**

EZH2 inhibition upregulates *BHMT* and *CDO1* expression through increasing *HNF4a* in HNF4α-negative mesenchymal cell. **A** *BHMT* and *CDO1* were suppressed in HCC tumors compared to non-tumor tissues from two independent cohorts. **B** Kaplan-Meier survival plots showing OS with high versus low expression of *BHMT* and *CDO1*. **C, D** RT-qPCR results showing derepression of *HNF4a*, *BHMT*, and *CDO1* by EZH2 inhibition in SNU-449 HNF4α-negative mesenchymal cell.

**Supplementary Fig. 6**

EZH2 inhibition downregulates ferroptosis negative regulator in HNF4α-negative mesenchymal cells. **A** Lipid peroxidation levels in HepG2 and Hep3B cells treated with siBHMT or siCDO1 (*n* = 3). **B** Cell viability in HepG2 and Hep3B cells treated with the indicated siRNAs and in the presence or absence of tazemetostat (*n* = 3). **C** RT-qPCR results showing expression levels of the ferroptosis negative regulator *SLC7A11* under tazemetostat treatment in SNU-449 cell. **D** *SLC7A11*, *NFS1,* and *FSP1* were overexpressed in HCC tumors compared to non-tumor tissues from TCGA cohorts.
