# Supplementary material for: Integration of epigenomic and transcriptomic profiling uncovers EZH2 target genes linked to cysteine metabolism in hepatocellular carcinoma
Source: Cell Death Dis. 2024 Nov 8;15(11):801. doi: 10.1038/s41419-024-07198-0 (PMC11549485; doi:10.1038/s41419-024-07198-0)
Supplement: Supplementary file 2 — Supplementary Figures [file 41419_2024_7198_MOESM2_ESM.pdf]

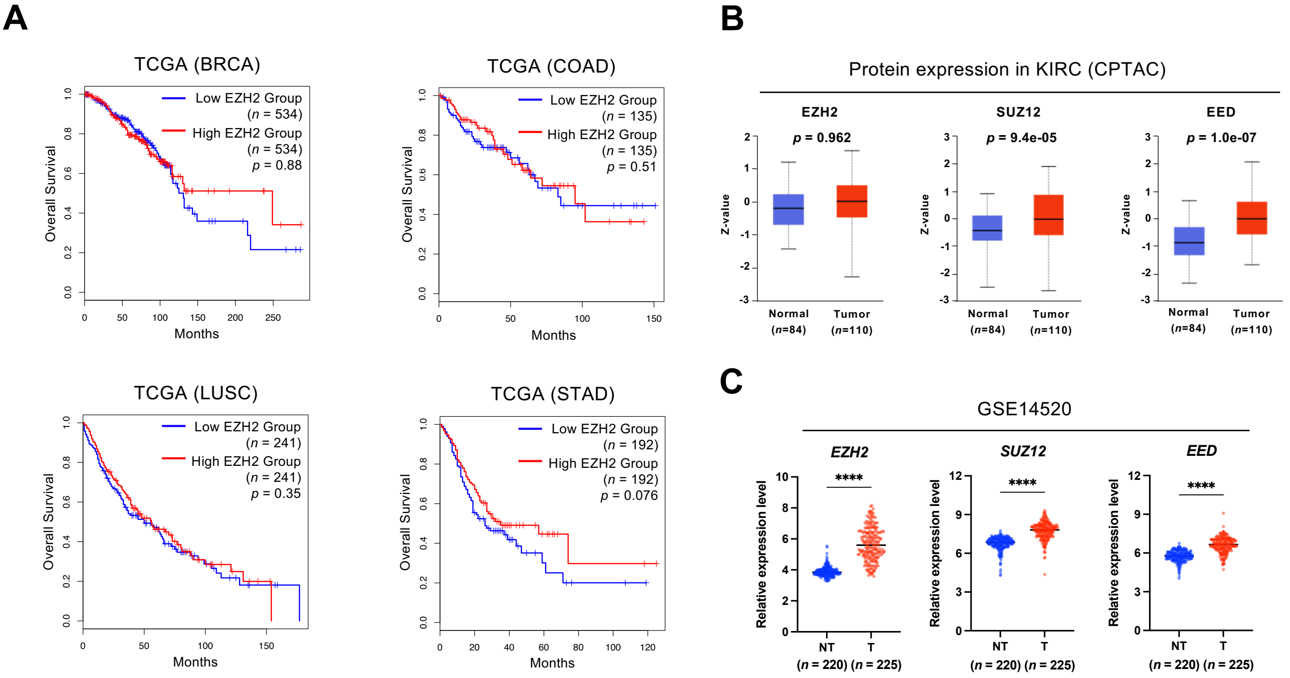
A
TCGA (BRCA)
Low EZH2 Group
(n = 534)
High EZH2 Group
(n = 534)
p = 0.88
Overall Survival
Months
TCGA (COAD)
Low EZH2 Group
(n = 135)
High EZH2 Group
(n = 135)
p = 0.51
TCGA (LUSC)
Low EZH2 Group
(n = 241)
High EZH2 Group
(n = 241)
p = 0.35
TCGA (STAD)
Low EZH2 Group
(n = 192)
High EZH2 Group
(n = 192)
p = 0.076
B
Protein expression in KIRC (CPTAC)
EZH2
p = 0.962
SUZ12
p = 9.4e-05
EED
p = 1.0e-07
Z-value
Normal
(n=84)
Tumor
(n=110)
C
GSE14520
EZH2
SUZ12
EED
****
Relative expression level
NT
T
(n = 220) (n = 225)

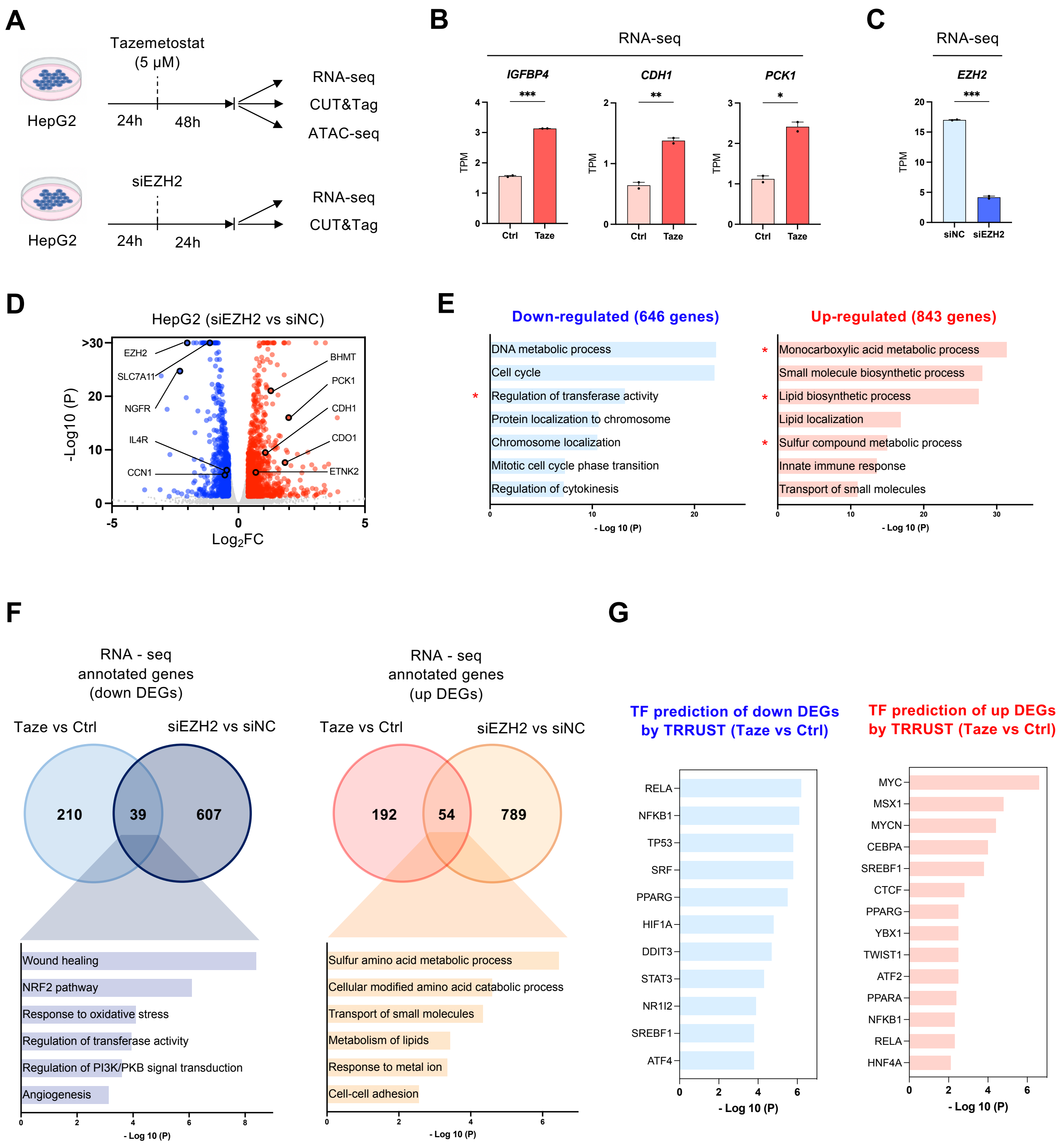
A
Tazemetostat
(5 μM)
HepG2
24h
48h
RNA-seq
CUT&Tag
ATAC-seq
siEZH2
HepG2
24h
24h
RNA-seq
CUT&Tag
B
RNA-seq
IGFBP4
CDH1
PCK1
TPM
Ctrl
Taze
C
RNA-seq
EZH2
siNC
siEZH2
D
HepG2 (siEZH2 vs siNC)
EZH2
SLC7A11
NGFR
IL4R
CCN1
BHMT
PCK1
CDH1
CDO1
ETNK2
-Log10 (P)
Log2FC
E
Down-regulated (646 genes)
DNA metabolic process
Cell cycle
Regulation of transferase activity
Protein localization to chromosome
Chromosome localization
Mitotic cell cycle phase transition
Regulation of cytokinesis
- Log 10 (P)
Up-regulated (843 genes)
Monocarboxylic acid metabolic process
Small molecule biosynthetic process
Lipid biosynthetic process
Lipid localization
Sulfur compound metabolic process
Innate immune response
Transport of small molecules
- Log 10 (P)
F
RNA - seq
annotated genes
(down DEGs)
Taze vs Ctrl
siEZH2 vs siNC
210
39
607
Wound healing
NRF2 pathway
Response to oxidative stress
Regulation of transferase activity
Regulation of PI3K/PKB signal transduction
Angiogenesis
- Log 10 (P)
RNA - seq
annotated genes
(up DEGs)
Taze vs Ctrl
siEZH2 vs siNC
192
54
789
Sulfur amino acid metabolic process
Cellular modified amino acid catabolic process
Transport of small molecules
Metabolism of lipids
Response to metal ion
Cell-cell adhesion
- Log 10 (P)
G
TF prediction of down DEGs
by TRRUST (Taze vs Ctrl)
RELA
NFKB1
TP53
SRF
PPARG
HIF1A
DDIT3
STAT3
NR1I2
SREBF1
ATF4
- Log 10 (P)
TF prediction of up DEGs
by TRRUST (Taze vs Ctrl)
MYC
MSX1
MYCN
CEBPA
SREBF1
CTCF
PPARG
YBX1
TWIST1
ATF2
PPARA
NFKB1
RELA
HNF4A
- Log 10 (P)

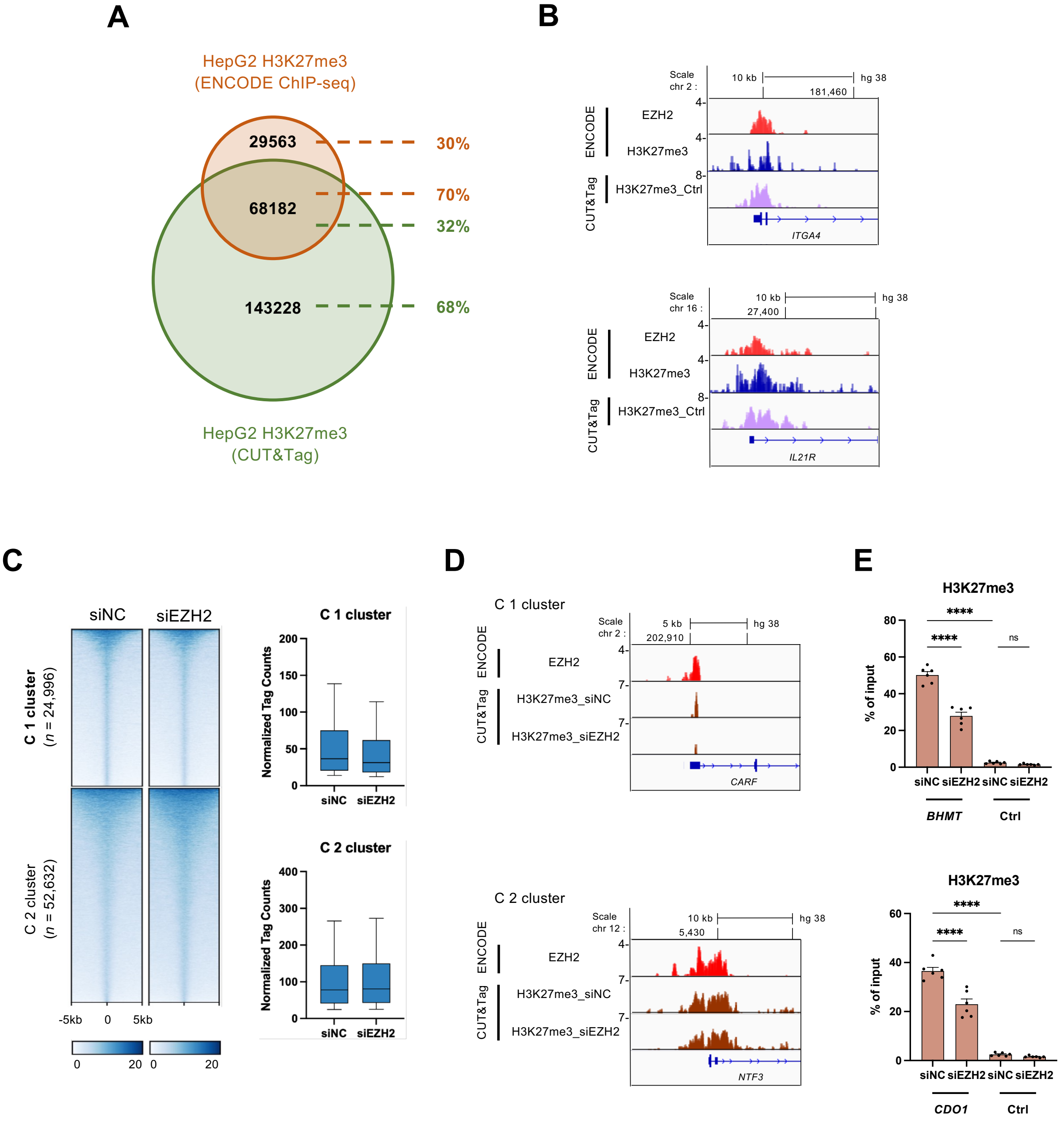

A
HepG2 H3K27me3
(ENCODE ChIP-seq)
29563
68182
143228
30%
70%
32%
68%
HepG2 H3K27me3
(CUT&Tag)
B
Scale
chr 2 :
10 kb
hg 38
181,460
ENCODE
EZH2
H3K27me3
CUT&Tag
H3K27me3_Ctrl
ITGA4
Scale
chr 16 :
10 kb
hg 38
27,400
ENCODE
EZH2
H3K27me3
CUT&Tag
H3K27me3_Ctrl
IL21R
C
siNC
siEZH2
C 1 cluster
(n = 24,996)
C 2 cluster
(n = 52,632)
-5kb
0
5kb
0
20
0
20
C 1 cluster
Normalized Tag Counts
siNC
siEZH2
C 2 cluster
Normalized Tag Counts
siNC
siEZH2
D
C 1 cluster
Scale
chr 2 :
5 kb
hg 38
202,910
ENCODE
EZH2
CUT&Tag
H3K27me3_siNC
H3K27me3_siEZH2
CARF
C 2 cluster
Scale
chr 12 :
10 kb
hg 38
5,430
ENCODE
EZH2
CUT&Tag
H3K27me3_siNC
H3K27me3_siEZH2
NTF3
E
H3K27me3
% of input
****
****
ns
siNC
siEZH2
siNC
siEZH2
BHMT
Ctrl
H3K27me3
% of input
****
****
ns
siNC
siEZH2
siNC
siEZH2
CDO1
Ctrl

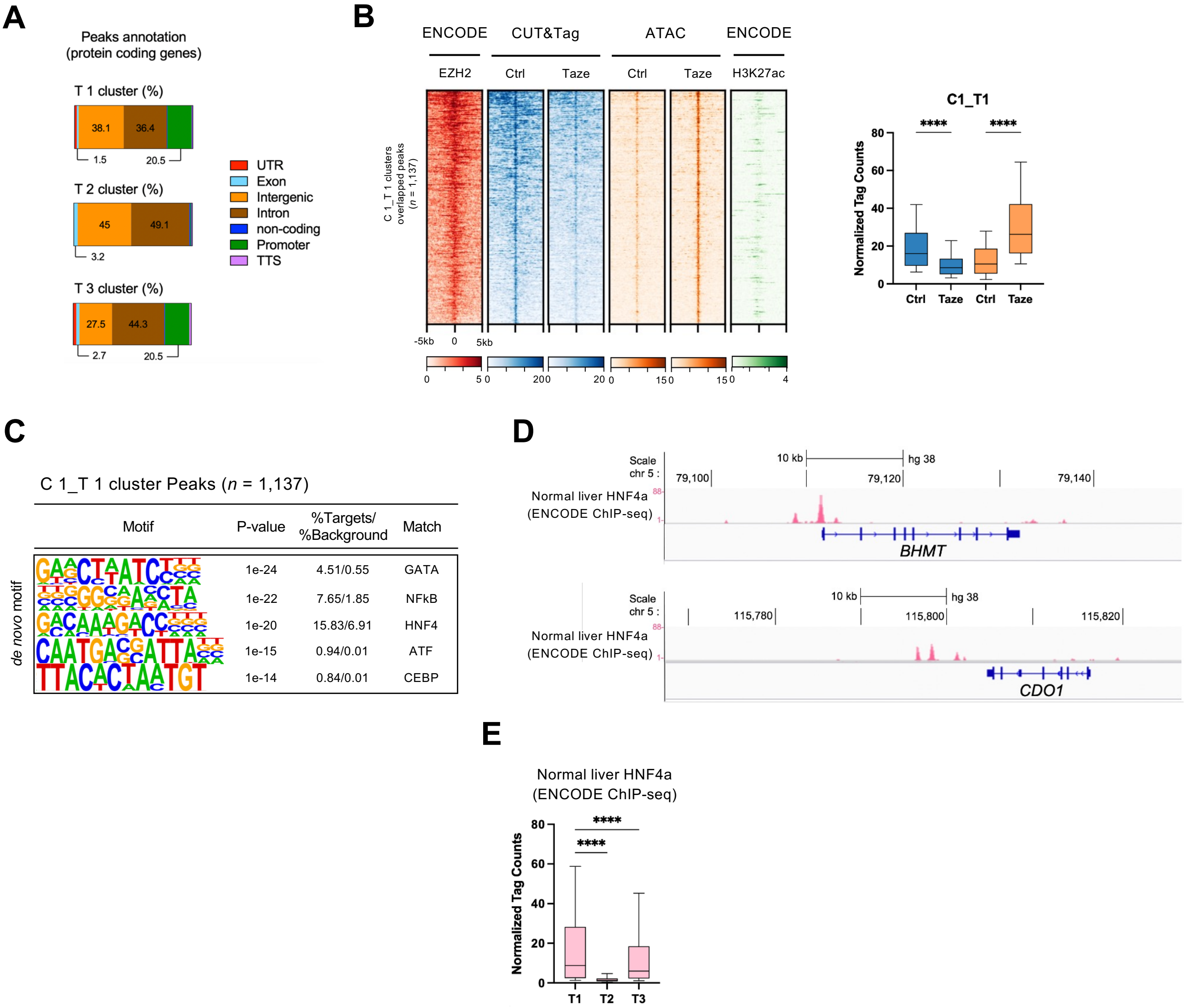
A
Peaks annotation
(protein coding genes)
T 1 cluster (%)
38.1
36.4
1.5
20.5
T 2 cluster (%)
45
49.1
3.2
T 3 cluster (%)
27.5
44.3
2.7
20.5
UTR
Exon
Intergenic
Intron
non-coding
Promoter
TTS
B
ENCODE
CUT&Tag
ATAC
ENCODE
EZH2
Ctrl
Taze
Ctrl
Taze
H3K27ac
C 1_T 1 clusters
overlapped peaks
(n = 1,137)
-5kb
0
5kb
0
50
200
20
0
150
150
4
C1_T1
****
****
Normalized Tag Counts
80
60
40
20
0
Ctrl
Taze
Ctrl
Taze
C
C 1_T 1 cluster Peaks (n = 1,137)
Motif
P-value
%Targets/
%Background
Match
de novo motif
1e-24
4.51/0.55
GATA
1e-22
7.65/1.85
NFkB
1e-20
15.83/6.91
HNF4
1e-15
0.94/0.01
ATF
1e-14
0.84/0.01
CEBP
D
Scale
chr 5 :
10 kb
hg 38
79,100
79,120
79,140
Normal liver HNF4a
(ENCODE ChIP-seq)
88
1
BHMT
Scale
chr 5 :
10 kb
hg 38
115,780
115,800
115,820
Normal liver HNF4a
(ENCODE ChIP-seq)
88
1
CDO1
E
Normal liver HNF4a
(ENCODE ChIP-seq)
****
****
Normalized Tag Counts
80
60
40
20
0
T1
T2
T3

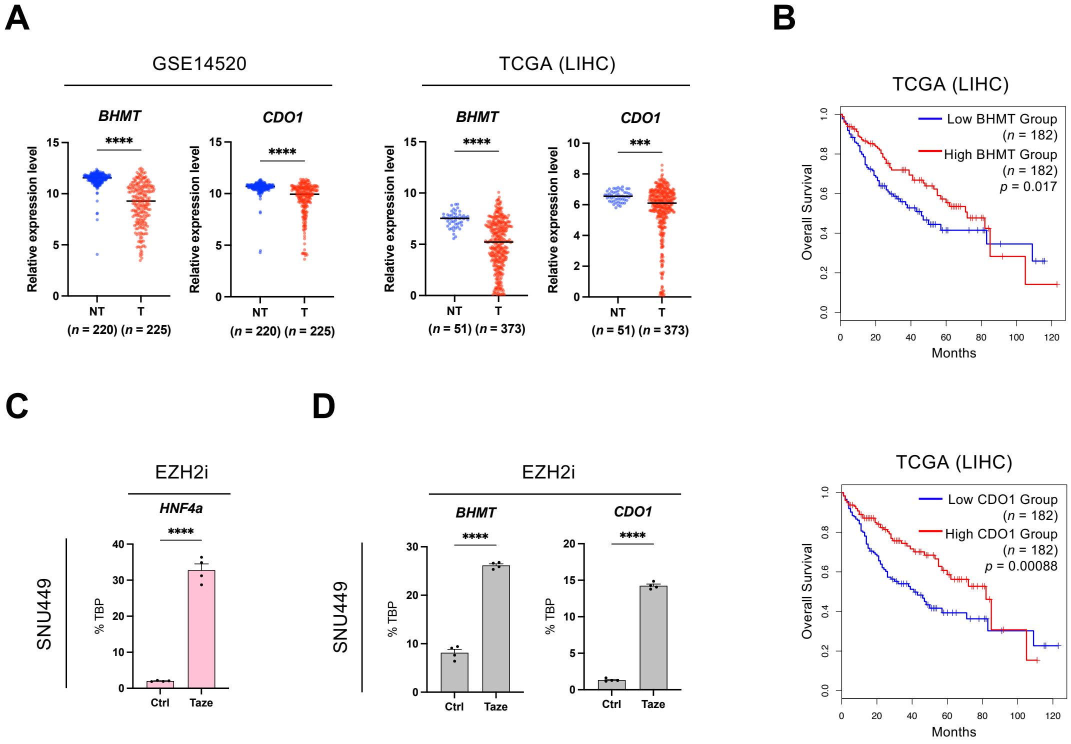
A
GSE14520
BHMT
CDO1
TCGA (LIHC)
BHMT
CDO1
Relative expression level
****
****
****
***
NT
T
(n = 220) (n = 225)
(n = 220) (n = 225)
(n = 51) (n = 373)
(n = 51) (n = 373)
B
TCGA (LIHC)
Low BHMT Group
(n = 182)
High BHMT Group
(n = 182)
p = 0.017
Overall Survival
Months
TCGA (LIHC)
Low CDO1 Group
(n = 182)
High CDO1 Group
(n = 182)
p = 0.00088
Overall Survival
Months
C
EZH2i
HNF4a
****
SNU449
% TBP
Ctrl
Taze
D
EZH2i
BHMT
CDO1
****
****
SNU449
% TBP
Ctrl
Taze

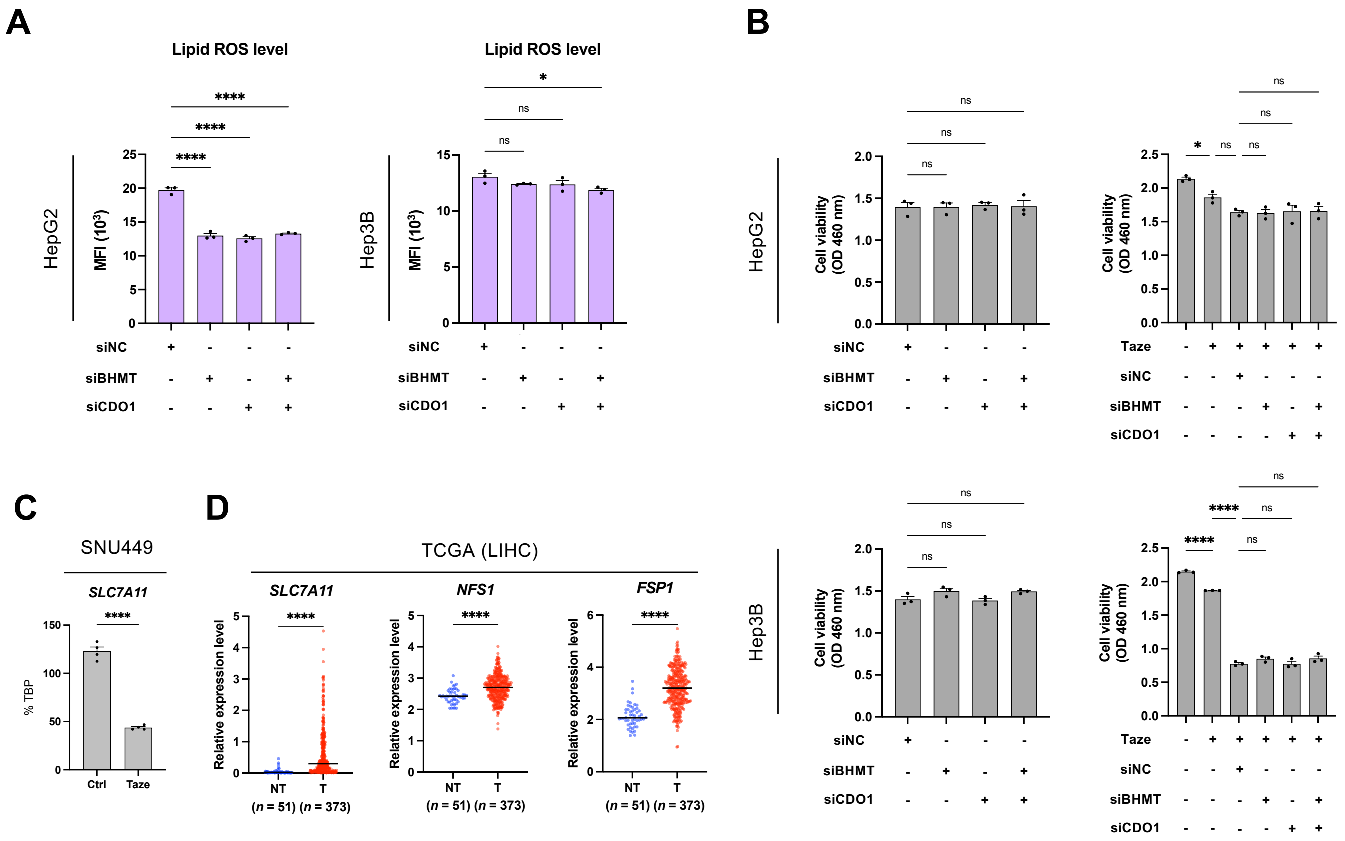
A
Lipid ROS level
HepG2
MFI (10³)
Hep3B
siNC
siBHMT
siCDO1
ns
*
****
B
Cell viability (OD 460 nm)
Taze
C
SNU449
SLC7A11
% TBP
Ctrl
D
TCGA (LIHC)
NFS1
FSP1
Relative expression level
NT
T
(n = 51) (n = 373)
