# Supplementary figures and images for: Integration of epigenomic and transcriptomic profiling uncovers EZH2 target genes linked to cysteine metabolism in hepatocellular carcinoma
Source: Cell Death Dis. 2024 Nov 8;15(11):801. doi: 10.1038/s41419-024-07198-0 (PMC11549485; doi:10.1038/s41419-024-07198-0)

Fig. 2A

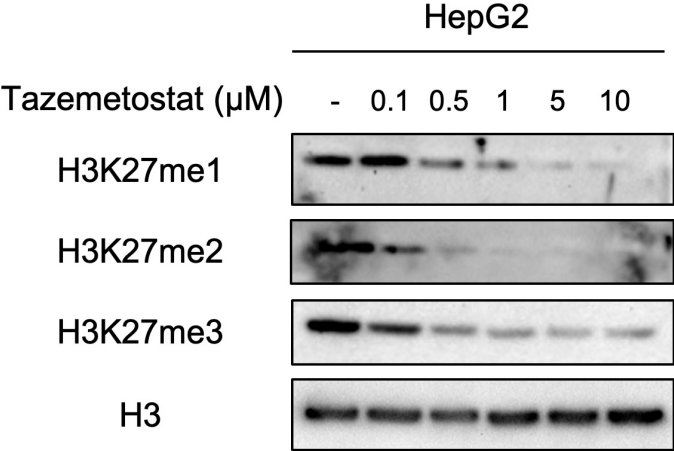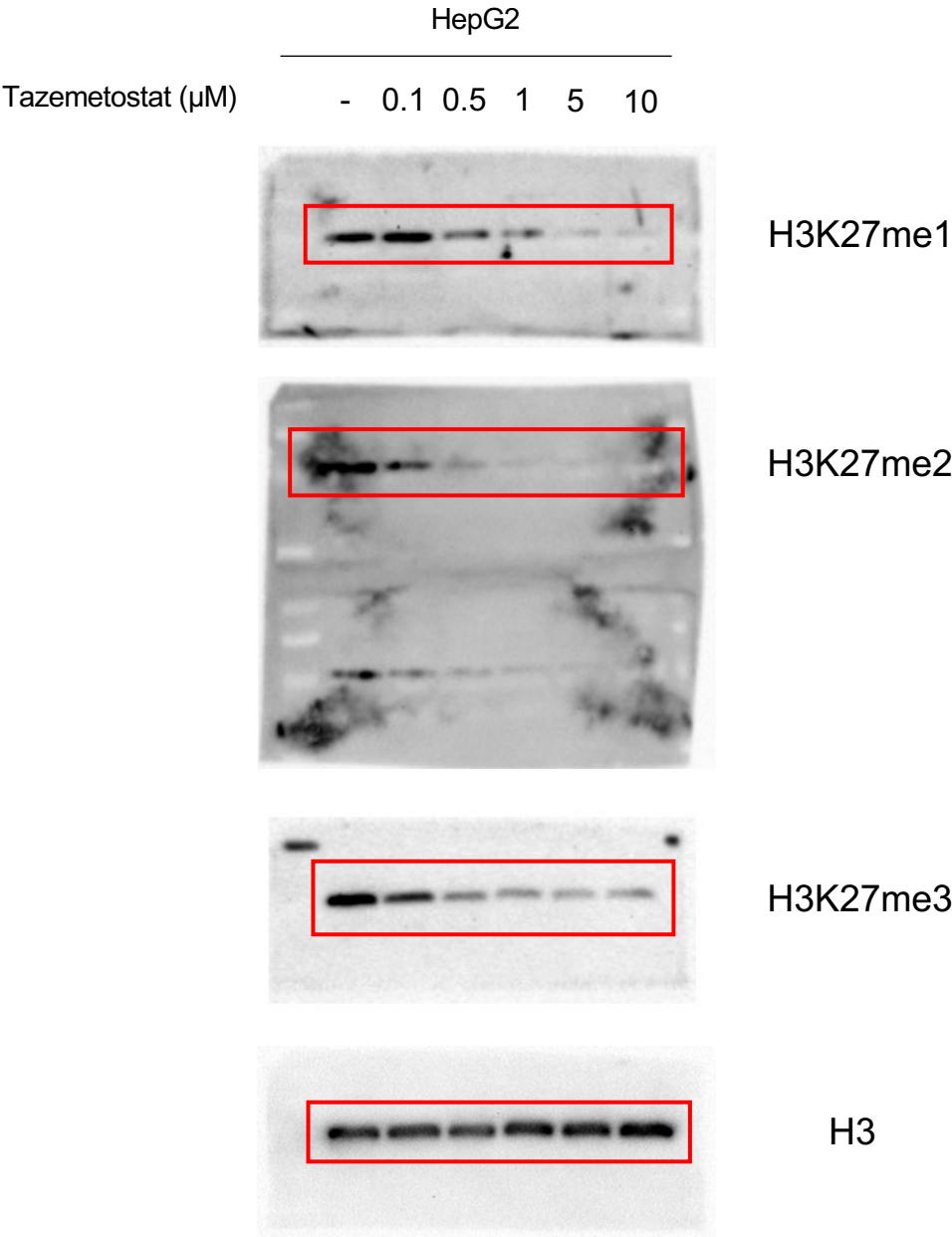

Fig. 2A

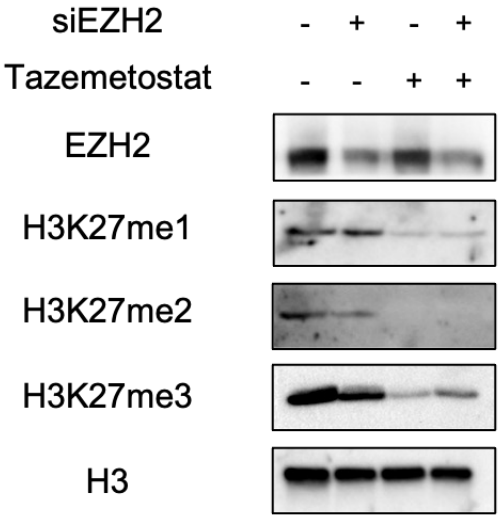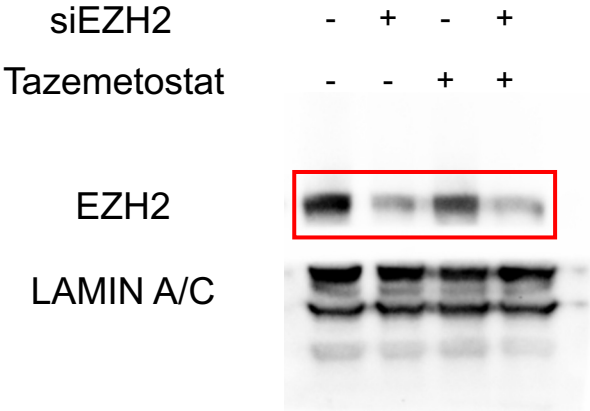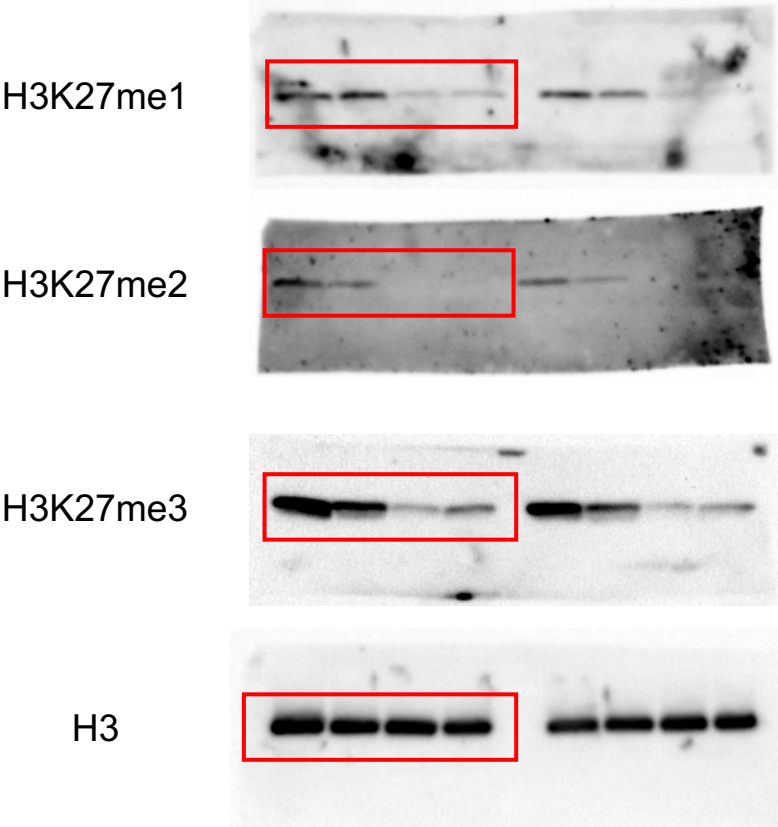

Supplement: Supplementary file 3 — Supplemental MateriaL [file 41419_2024_7198_MOESM3_ESM.pdf]
